# The Best Models of Bodipy’s Electronic Excited State: Comparing Predictions from Various DFT Functionals with Measurements from Femtosecond Stimulated Raman Spectroscopy

**DOI:** 10.1021/acs.jpca.3c05040

**Published:** 2023-09-26

**Authors:** Juan S. Sandoval, David W. McCamant

**Affiliations:** Department of Chemistry, University of Rochester, 120 Trustee Road, Rochester, New York 14627, United States

## Abstract

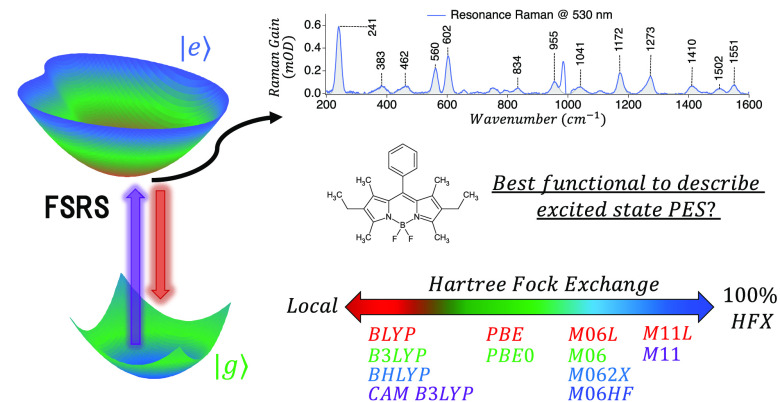

Density functional theory (DFT) and time-dependent DFT (TD-DFT) are pivotal approaches for modeling electronically excited states of molecules. However, choosing a DFT exchange-correlation functional (XCF) among the myriad of alternatives is an overwhelming task that can affect the interpretation of results and lead to erroneous conclusions. The performance of these XCFs to describe the excited-state properties is often addressed by comparing them with high-level wave function methods or experimentally available vertical excitation energies; however, this is a limited analysis that relies on evaluation of a single point in the excited-state potential energy surface (PES). Different strategies have been proposed but are limited by the difficulty of experimentally accessing the electronic excited-state properties. In this work, we have tested the performance of 12 different XCFs and TD-DFT to describe the excited-state potential energy surface of Bodipy (2,6-diethyl-1,3,5,7-tetramethyl-8-phenyldipyrromethene difluoroborate). We compare those results with resonance Raman spectra collected by using femtosecond stimulated Raman spectroscopy (FSRS). By simultaneously fitting the absorption spectrum, fluorescence spectrum, and all of the resonance Raman excitation profiles within the independent mode displaced harmonic oscillator (IMDHO) formalism, we can describe the PES at the Franck–Condon (FC) region and determine the solvent and intramolecular reorganization energy after relaxation. This allows a direct comparison of the TD-DFT output with experimental observables. Our analysis reveals that using vertical absorption energies might not be a good criterion to determine the best XCF for a given molecular system and that FSRS opens up a new way to benchmark the excited-state performance of XCFs of fluorescent dyes.

## Introduction

Density functional theory (DFT) and its extension, time-dependent (TD) DFT, have become foundational tools for modeling electronically excited states and supporting experimental studies. The low computational cost makes it possible to investigate the excited states of large molecular systems in the condensed phase that would be impossible to model with high-level wave function (HLWF) methods. Even though DFT is, in principle, an exact approach,^1−3^ its accuracy is primarily determined by the exchange-correlation functional (XCF), which contains all the approximations of the model and is where most of the efforts to improve DFT/TD-DFT are oriented.^3,4^

Two of the strategies to improve the performance of XCF’s are the Generalized Gradient Approximation (GGA) and the meta-GGA (mGGA). The former includes the explicit dependence on the electronic density and its gradient, and the latter adds the kinetic energy of the electronic density. Perhaps the most remarkable steps toward refining the XCF are the hybrid functionals, which include nonlocal Hartree–Fock exchange (HFX).^4^ This term exactly cancels the self-interaction energy (SIE) of the Coulombic integral in the Hartree–Fock formulation.^5,6^ Given their success in describing molecular properties in the ground state, plenty of hybrid functionals are available that differ in the percentage of HFX incorporated and its dependence on the interelectronic distance.^7^ Global hybrid (GH) XCFs have a constant HFX contribution, while range-separated hybrid (RSH) XCFs have a different contribution of HFX for short and long interelectronic distances. Regardless of the extraordinary progress in this field and the emergence of newer XCFs, it is still a challenge to determine the “correct” XCF to use without testing its performance. A universally correct inclusion of HFX into the XCFs is yet to be determined.

Extensive work has been performed to benchmark different XCFs for their ground- and excited-state properties. The two strategies adopted for this purpose are (i) a direct comparison with HLWF methods, a theory-to-theory approach, and (ii) a comparison with accurate experimental data.^6,8^ The former allows an equal footing comparison of properties calculated under the same conditions; however, it is limited by the availability of theoretical data, i.e., small to medium size molecules. HLWF methods, such as CC3 and full CI, become hopeless for molecular systems with more than 20 atoms due to their scaling with molecular size.^9^ Conversely, the latter approach allows for comparing the performance of different XCFs with large “real-life” molecules, but it can be limited by the kinds of experimental data that are broadly available.

Commonly, studies devoted to benchmarking different XCFs compare the vertical absorption energies, defined as the energy difference of the ground and excited electronic states at the optimized ground state geometry, of several excited states versus the HLWF methods or experiments.^6−8,10−13^ This tests a single point in the excited-state potential energy surface (PES), and a proper comparison with the experimental observable, the “0–0” energy (*E*_00_^*Abs*^), would require vibrational calculations which could be prohibited in some HLWF methods.^14^ Moreover, the absorption measurements are often performed in solution, requiring further approximations.^15^ Additionally, in photochemistry and photophysics, we are interested in the shape of the lowest (or lower) excited-state PESs, which determines the excited-state dynamics,^16^ rather than the accuracy of depicting high-energy states or even Rydberg states, which are often used to benchmark gas-phase molecules.

Another approach to benchmarking the excited-state performance of XCFs, which goes beyond the simple vertical energy comparison, is reproducing the electronic spectra’s vibronic structure,^17−19^ providing insights on the excited-state PES. Typically, this has been performed by calculating a molecule’s vertical, adiabatic, and 0–0 energy transition energies.^7,8,14,15,18,20−24^ For similar reasons, vertical emission is preferred over vertical absorption, since an accurate estimation of the emission energy requires a good description of the excited-state PES.^25^ The oscillator strength (*f*_*osc*_^*Abs*^) and excited-state dipole moments have also been proposed as a metric to benchmark the performance of XCFs in the excited state.^23,26−29^ A correct estimation of *f*_*osc*_^*Abs*^ requires an accurate description of the ground and excited electronic densities and the transition dipole moment.^27^ However, when calculating the zero-point energy and excited-state reorganization energy, that is, the difference between the vertical and adiabatic transition energies, the particular coupling of each normal mode is hidden in the calculation, which only reports the total reorganization energy rather than the reorganization energy magnitude along each normal mode vector.^24,30^ In other words, a method could predict the correct magnitude of the energy gap as the molecule relaxes from the Franck–Condon point to the equilibrium point on the excited-state surface, but the direction of that motion might be incorrect. Hence, a new approach that explicitly maps out the excited-state PES by separating out the excited-state forces into projections along each normal mode is still required to test the performance of the TDDFT methods.

Resonance Raman (RR) spectroscopy is a probe for the excited-state PES,^31,32^ and within the independent mode displaced harmonic oscillator (IMDHO) model formalism, it is possible to relate the RR excitation profile with structural changes in the Franck–Condon region^33−36^ (FC), thus mapping out the shape of the excited-state PES after photoexcitation. For any RR spectrum, the frequencies of the observed modes are determined by the ground-state PES, but the intensities of the RR peaks are determined by the displacements of each normal mode in the excited state, i.e., how coupled each normal mode is to the resonant electronic transition. This coupling can be calculated by projecting the atomic forces in the Franck–Condon point onto the normal modes of the ground state.^37^ Additionally, details of the electronic dephasing and the multidimensional nature of the excited-state motion play a critical part in determining the intensity of each peak in the spectrum.^31,32^

We employ femtosecond stimulated Raman spectroscopy^38,39^ (FSRS) to collect the ground state RR at different laser frequencies and construct the RR excitation profile. FSRS allows the collection of RR spectra of strongly fluorescent molecules that have previously been inaccessible to RR analysis and can provide, for the first time, a window into the multidimensional structural reorganization occurring in these molecules after the absorption of light.

Prior work, particularly by Elles and co-workers, has directly compared FSRS spectra of both the ground-state and excited states with spectra calculated by TD-DFT.^40−42^ Much of that impressive work used nonresonant Raman spectra calculated with TD-DFT to assign the observed Raman spectra of both the ground- and excited-state species and was not focused on determining the best functionals for the purpose. In 2018, Quincy et al. required the use of EOM-CCSD theory to reasonably match the RR spectrum of the *S*_1_ state of 2,5-diphenylthiophene (DPT) when B3LYP TD-DFT failed to predict the correct relative intensities.^41^ But the theoretical spectra consistently showed weak intensities in the low-frequency region, up to 25 times smaller than the experiment, which may have been due to the simplified implementation of RR theory to convert the theoretical energy gradients into RR intensities. In this work, we apply state-of-the-art resonance Raman theory to fully analyze the experimental resonant-stimulated Raman spectra and to convert the parameters calculated by TD-DFT to RR spectra. We hope that these higher-level Raman intensity calculations will allow a more direct comparison of the relative intensities predicted by various exchange-correlation functionals.

**Figure 1:**
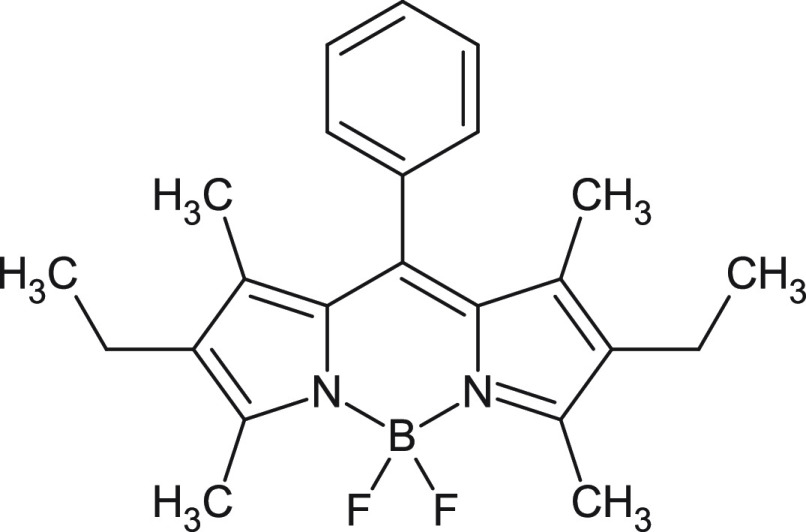
Bodipy: 2,6-Diethyl-1,3,5,7-tetramethyl-8-phenyldipyrromethene difluoroborate.

In this work, we use 2,6-diethyl-1,3,5,7-tetramethyl-8-phenyldipyrromethene difluoroborate (Bodipy, Figure 1) as a model system to benchmark the accuracy of different XCFs and TD-DFT to model electronically excited states that rely on the multidimensional evaluation of the energy gradient at the FC region and not on a single point on the excited-state PES. Additionally, including the Brownian oscillator solvation model^43^ in the optical line shape modeling makes it possible to account for the solvent and intramolecular contribution to the total reorganization energy, allowing a direct comparison of the DFT output and the experimental observables.

## Methods

### Steady-State Spectroscopy

2,6-Diethyl-1,3,5,7-tetramethyl-8-phenyldipyrromethene difluoroborate (Bodipy) was purchased from Sigma-Aldrich. Molar absorptivity was determined from absorption spectra collected in a Shimadzu UV-1800 spectrophotometer using a 1 mm fused-silica cuvette, and the emission spectra were collected in a Spex Fluoromax-3 fluorometer (Horiba) with a photomultiplier tube detector. The sample absorbance was kept below 0.1 OD in fluorescent experiments. The excitation wavelength was set to 527 nm.

### Femtosecond Stimulated Raman Spectroscopy

Raman pump pulses (541, 530, and 516 nm) were obtained after spectral filtering, by a 4*f* filter, the output of a home-built noncollinear optical parametric amplifier (NOPA) pumped by the frequency-doubled output of a regenerative amplified Titanium:sapphire laser system (Spectra-Physics, 800 nm, 1 kHz). The NOPA output had a bandwidth of ∼10 nm, obtained by adding 1 cm of water to the white-light seed path to additionally chirp it before amplification. Then the NOPA output was then dispersed by an 1800 gr/mm holographic grating and focused by a 300 mm focal length spherical lens through a ∼0.5 mm slit. The resulting pulses had a bandwidth <20 cm^–1^, and a pulse duration of ∼1.6 ps.

The 480 nm Raman pulse was generated by focusing a 400 nm pulse through a Raman shifter filled with pressurized (850 psi) H_2_ gas. The details of this setup can be found elsewhere.^44,45^ Briefly, the intense 400 nm 2.5 ps pulse is generated by the sum of frequency generation of two oppositely chirped 800 nm pulses obtained through a home-built second-harmonic-bandwidth compressor (SHBC). Then, the 400 nm pulse overlaps with a chirped white-light continuum in the 50 cm H_2_ pipe. The Raman shifter output consisting of five different wavelengths (300, 343, 400, 480, and 600 nm) is dispersed by a prism, and the 480 nm wavelength is selected with a slit. The Raman pulse duration was ∼2.5 ps with a spectral bandwidth of 11 cm^–1^.

The FSRS white-light probe was produced by focusing the 800 nm fundamental into a 2 mm sapphire crystal to produce a continuum from 420 to 900 nm. The remaining 800 nm fundamental was blocked before the sample by a near IR absorbing dye solution (NIR800A, QCR Solutions Corp). The Raman pump pulses were chopped at 500 Hz. After the sample, the probe was dispersed by a 600 grooves/mm grating in second-order and focused onto a charge-coupled device camera (Princeton Instruments Pixis 100BR). The scanning multichannel technique (SMT) increased the signal-to-noise ratio by eliminating the systematic noise pattern.^46^ The Raman signal is shifted by 1 pixel 20 times and averaged for 2 s at each position. This process is repeated 10 times for a total time of 6.67 min. All spectra were collected using a 2 mm quartz cuvette, and the Raman shift axis was calibrated to that of cyclohexane.

### Experimental Cross Sections

We collected RR spectra at different frequencies within the absorption band of Bodipy (541, 530, 516, and 480 nm) to construct the RR excitation profile. The raw data and the baseline subtracted FSRS spectra are presented in Figures S1 and S2, respectively. Absolute RR cross sections for the mode *k* were obtained as follows:^33,47,48^
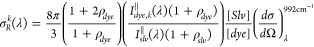
1in which “*dye*” refers to the parameters of the dye (solute) and “*slv*” are those of the solvent. Raman spectra were collected at parallel Raman pump–probe polarizations, *I*^∥^, using the benzene 992 cm^–1^ peak (*I*_*slv*_^∥^) as the internal standard. The solute’s intensities were obtained after fitting the baseline corrected FSRS spectra to a sum of Gaussians and integrating the area of the *k* peak (*I*_*dye*,*k*_^∥^) associated with vibrational mode *k*. ρ_*slv*_ is the depolarization ratio of the 992 cm^–1^ benzene mode, equal to 0.02,^49^ and ρ_*dye*_ is the depolarization ratio for each solute peak, set to 0.33. The differential cross sections (*dσ*/*d*Ω) of the 992 cm^–1^ benzene peak at each Raman pump wavelength, extrapolated from the literature,^49^ were 2.39 (541 nm), 2.60 (530 nm), 2.96 (516 nm), and 4.13 (480 nm) × 10^–13^ Å^2^/molecule.

Absorption cross section (Å^2^/molecule) was obtained by converting the molar absorptivity using the following relation:^31^
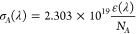
2Where ε(λ) is the extinction coefficient (M^–1^ cm^–1^), and *N*_*A*_ is Avogadro’s number.

### Time-Domain Methods for Computing Absolute Cross Sections

Theoretical line shape analysis to compute resonance Raman and absorption cross section have been extensively discussed in the literature.^31,32,48,50^ We outline the most relevant aspects necessary to discuss the present work in the SI. A complete description of the theoretical model and the code implemented in this work to simulate optical line shapes can be found in ref (48). Briefly, the excited-state displacements of every mode, Δ_*k*_, are adjusted, along with the excited-state energy, *E*_0_, and homogeneous and inhomogeneous line widths, Γ and θ, and transition dipole length, μ, to simultaneously fit the absorption spectrum, fluorescence spectrum, and all *k* RREPs.

By choosing the Brownian oscillator model^43^ to represent the solute–solvent interactions (details can be found in the SI), it is possible to determine the solvent contribution (*RE*_*s*_) from the total reorganization energy (*RE*_*T*_), as follows:
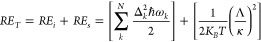
3where *RE*_*i*_ corresponds to the intramolecular reorganization energy assuming harmonic PESs. Here, *N* is the total number of normal modes, Λ is the modulation frequency, *K*_*B*_ is the Boltzmann constant, and κ = Λ/*D* the solvent parameter, which is related to the full-width half maxima of the line shape, Γ, an adjustable parameter during the fitting process, and *D* is the solvent coupling strength

To determine the set of parameters that best fit the experimental absorption and emission spectra and all the *N* resonance Raman profiles, we augmented the code implemented previously by our group.^48^ The search of the *N* + 4 parameters (*N* Δ’s for the *N* modes, *E*_0_, Γ, θ, and μ) is semiautomatized, and the chosen values result from the minimization of the error between the experimental and modeled absorption and Raman cross sections. The initial guess for μ comes from integrating the absorption spectra.^51^*E*_0_ can be approximated by the energy at the crossing point between the normalized absorption and emission spectra. The fwhm of the absorption can be used as an initial guess for Γ, and θ is adjusted after all parameters are optimized. The RR experiment defines the set of vibrational frequencies {ω_*k*_}, and the initial guess for the FC displacements  is obtained from their relative intensities at 541 nm, an excitation wavelength where low-frequency modes are present. Then, the algorithm adjusts  until the error is minimized, ending the first iteration. Following this, *E*_0_, Γ, and μ are manually fine-tuned, and a new iteration starts using , obtained after the first iteration. The process ends when no better solution can be found.

The theoretical framework outlined here, in combination with FSRS and steady-state spectroscopy, makes it possible to access molecular information and directly compare it to the TD-DFT/DFT outputs.

### Electronic Structure Methods

All electronic calculations presented in this work were performed in the Gaussian 16 software package.^68^ The list of all XCFs used in this work, including their types and HFX percentages, is presented in Table 1. We employed the ChemCraft^69^ program to visualize the molecular and natural transition orbitals^70^ (NTO).

**Table 1:**
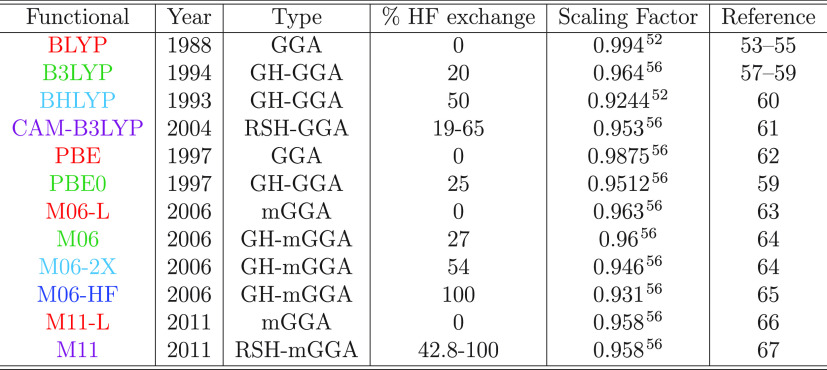
List of Exchange-Correlation Functionals Used in This Work^52−67^

The Bodipy dye was initially optimized in Avogadro^71^ using the universal force field (UFF) and later using DFT at the B3LYP^57−59^/6-311G* level of theory (LOT). Then, this structure is used as input for the ground state optimization and frequency calculation for each XCF. We used the well-known polarizable continuum model^72^ (PCM) to include solvent effects. We chose benzene for every ground and excited-state calculation, since it was the solvent used to collect RR, absorption, and emission spectra.

**Figure 2:**
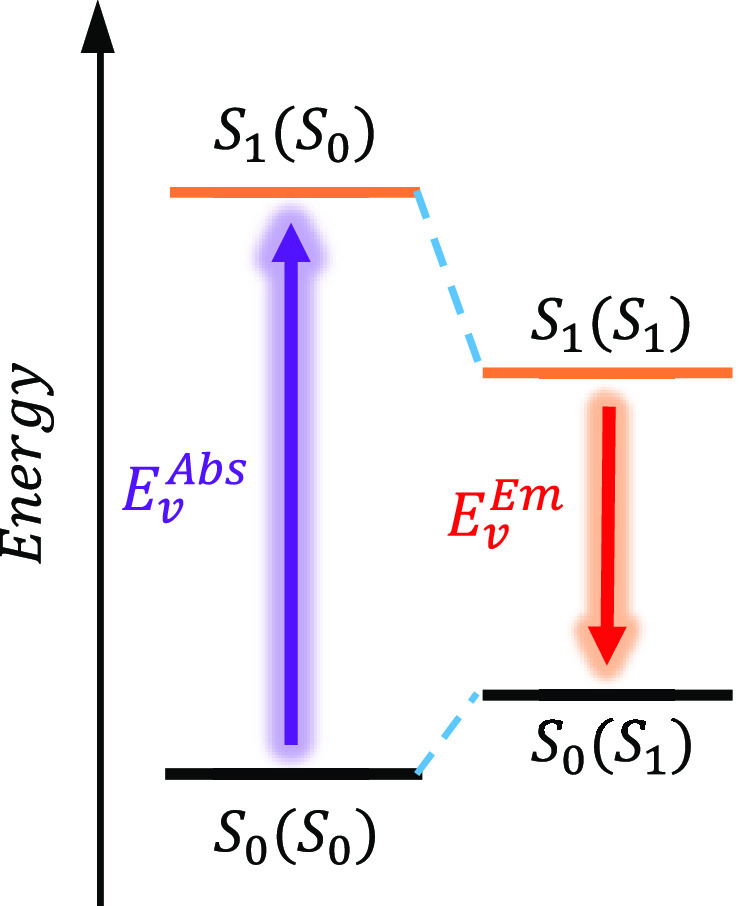
Calculation of the vertical absorption (*E*_*v*_^*Abs*^) and emission (*E*_*v*_^*Em*^) energies using DFT/TD-DFT and the SS-PCM formalism. Absorption is initiated from the ground electronic state at the equilibrium geometry and solvent polarization of the ground state, *S*_0_(*S*_0_), and places the molecule in the excited electronic state but with the nuclear geometry and solvent polarization of the ground state, *S*_1_(*S*_0_). Emission is initiated at the excited-state equilibrium geometry and solvent polarization of the excited state, *S*_1_(*S*_1_), and places the molecule in the ground state but with the nuclear and solvent polarization of the excited state, *S*_0_(*S*_1_).

We employed TD-DFT for calculating the excited-state properties of the Bodipy dye for all 12 XCFs considered in this work. Vertical absorption (*E*_*v*_^*Abs*^) and emission (*E*_*v*_^*Em*^) energies were computed in the state-specific (SS) formalism^73^ within the PCM/TD-DFT method (SS-PCM/TD-DFT), and excited-state geometry optimizations were performed at the linear-response (LR) PCM/TD-DFT level. As shown in Figure 2, *E*_*v*_^*Abs*^ was calculated as the energy difference between the ground electronic state at optimized ground state geometry *S*_0_(*S*_0_) and the excited-state energy at ground state geometry or *S*_1_(*S*_0_). Here, the *S*_1_(*S*_0_) is in the solvent nonequilibrium (NEQ) regime, where the solvent fast degrees of freedom (DOFs) are in equilibrium with the excited-state electronic density, and the slow solvent DOFs are equilibrated at the ground state electronic density.^73^*E*_*v*_^*Em*^ is calculated as the difference between (i) the energy of the *S*_1_ state at the equilibrium geometry, *S*_1_(*S*_1_), within the equilibrium solvation regime (EQ), where the full solvent DOFs are in equilibrium with the excited-state electron density, and (ii) the ground state energy within the NEQ solvation regime at the *S*_1_ optimized geometry, *S*_0_(*S*_1_). Finally, the DFT total reorganization energy is defined as
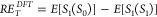
4where *E*[*S*_1_(*S*_0_)] and *E*[*S*_1_(*S*_1_)] correspond to the energy at *S*_1_(*S*_0_) and *S*_1_(*S*_1_), respectively.

Dimensionless displacements ({Δ_*k*_}) were calculated using the energy gradient of the excited-state PES at the ground state geometry (force calculation), and the ground state normal vectors were calculated at the optimized ground state geometry. The energy gradient comes from the excited state with the largest oscillator strength, *f*_*osc*_^*Abs*^. The basis set for all of the calculations was 6-311G*, and the geometry convergence for every calculation was set to *tight* and the grid to *ultrafine*.

### Exchange-Correlation Functionals Studied

The Minnesota meta-functionals stand out in the long list of hybrids developed in the last decades. It has shown remarkable success in describing excited-state properties, particularly the energy of the charge-transfer states. The M06 family functionals comprise four members (M06-L, M06, M06-2X, and M06-HF) with similar functional forms but different HFX contributions. In addition, we include M11-L and M11, the first RSH Minnesota functional developed. For comparison, we include perhaps the most widely used functionals BLYP, BHandHLYP (BHLYP), B3LYP, and CAM-B3LYP. This second set of GGA functionals includes GHs with different percentages of HFX and one RSH, CAM-B3LYP. This group of functionals allows testing the relevance of the inclusion of HFX to describe excited-state properties and also testing the accuracy of meta-GGA over GGA functionals.

TD-DFT calculations were performed using 12 XCFs of different kinds, Table 1. We included 6 GGA (BLYP, B3LYP, BHLYP, PBE, PBE0, and CAM-B3LYP) and 6 mGGA (M06-L, M06, M06-2X, M06-HF, M11-L, and M11) XCFs. Among them, we have four XCFs that do not include HFX (BLYP, PBE, M06-L, and M11-L), also known as “local” functionals, and two of them (CAM-B3LYP and M11) are RSH. A complete description of these methods and parameters can be found in the citations presented in Table 1.

Throughout this work, we color-label the XCFs according to the HFX included, as follows:

- The local functionals (BLYP, PBE, M06-L, and M11-L) are shown in red.
- GH functionals that include 20–30% of HFX are colored in green (B3LYP, PBE0, and M06).
- GH functionals with 50–60% of HFX are in light blue (BHLYP and M06-2X).
- M06-HF is the only XCF with 100% HFX, colored in dark blue.
- RSH functionals are colored in violet (CAM-B3LYP and M11).

**Table 2:** Experimental Raman Frequencies and Displacements Used to Fit the Absorption Spectra, Emission Spectra, and RR Excitation Profiles of Bodipy

Frequency (cm^–1^)	Δ
241	0.77
383	0.34
462	0.23
560	0.41
602	0.44
653	0.12
699	0.1
752	0.13
795	0.12
834	0.16
955	0.23
1041	0.18
1111	0.12
1172	0.34
1255	0.2
1273	0.24
1410	0.21
1458	0.1
1502	0.18
1551	0.21

## Results and Discussion

### Experimental and Modeled Cross Section

**Figure 3:**
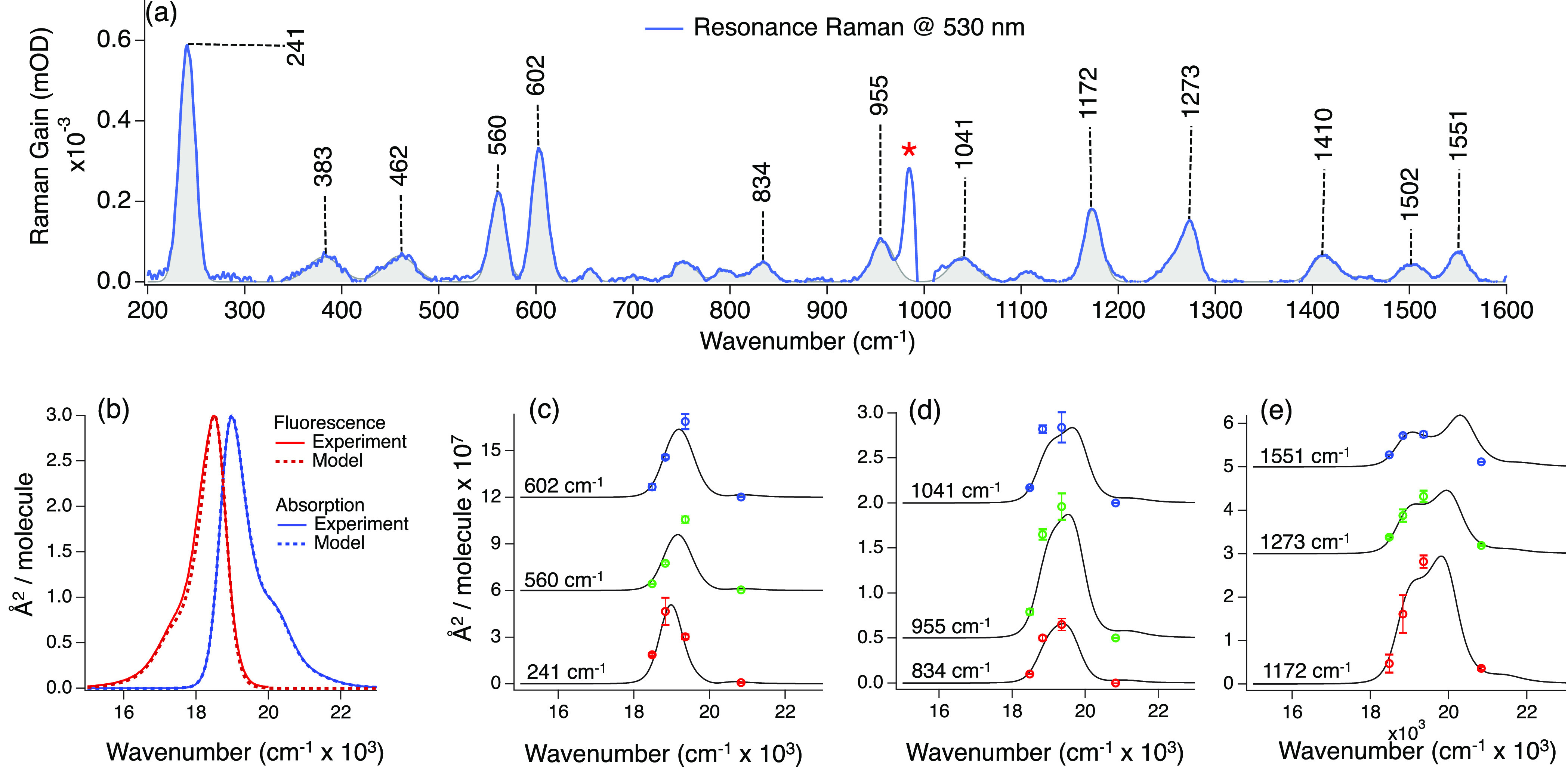
(a) 530 nm Raman pump FSRS spectrum (blue) and fit used to compute the Raman cross section (gray). The asterisk marks a residual feature from solvent subtraction. (b) Experimental absorption and emission spectra and the calculated absolute cross sections. Experimental and calculated resonance Raman cross section of the most intense vibrational modes in the (c) low-, (d) mid-, and (e) high-frequency region at each Raman pump wavelength. The calculated Raman cross sections are presented as solid lines.

Figure 3 presents the RR spectrum of Bodipy collected at 530 nm along with the absorption spectra, emission spectra, and the RR excitation profiles (RREP). In Figure 3b–d, we include the resulting fit that best describes the experimental data. The fit is obtained by adjusting the Δs, the electronic energy gap *E*_0_, the homogeneous (Γ) and inhomogeneous (θ) broadening, and the transition dipole length (μ) until the theoretical line shape simultaneously reproduced the absorption, emission, and all RREPs satisfactorily. The experimental resonance Raman cross sections shown in Figure 3 are presented in Table S1.

Through RR and theoretical line shape functions, we mapped out the multidimensional energy gradient of the excited-state PES at the FC region. Similarly, the Brownian oscillator model (implemented in the source code) allows separation of the solvent contribution from the total reorganization energy. The molecular parameters and the reorganization energy (eq 3) established after fitting are shown in Tables 3 and 4.

**Table 3:** Spectroscopic Parameters for Bodipy Were Established from the Fitting

Adjustable Parameters	
Electronic Energy Gap, *E*_0_ (cm^–1^)	18 730
Homogeneous Broadening, Γ (cm^–1^)	620
Inhomogeneous Broadening, θ (cm^–1^)	<50
Electronic transition dipole length, μ (Å)	1.781

**Table 4:** Reorganization Energy from Fitting and Experimental Stokes Shift

Reorganization Energy	
Intramolecular (cm^–1^)	520
Solvent (cm^–1^)	173
Total (cm^–1^)	693
Stokes Shift (cm^–1^)	499

### Steady-State Methods to Benchmark the Excited-State Performance of XCFs

**Figure 4:**
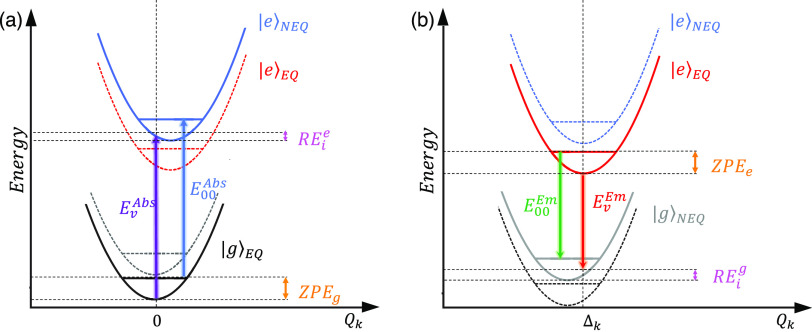
Ground (|*g*⟩) and excited (|*e*⟩) potential energy surfaces within the IMDHO model. The |*e*⟩ is presented at two different solvent regimes: equilibrium (*eq*), in red, and nonequilibrium (*neq*), in blue. Transitions energies benchmarked in this work: the vertical absorption energy, *E*_*v*_^*Abs*^ (purple), and the vertical emission energy, *E*_*v*_^*Em*^ (green), are obtained directly from TD-DFT. *ZPE*_*g*_ and *ZPE*_*e*_ are the zero-point vibrational energies for the ground and excited state, respectively. Similarly, *RE*_*g*_^*int*^ and *RE*_*e*_^*int*^ are the intramolecular reorganization energy for the ground and excited state. *E*_00_^*Abs*^ and *E*_00_^*Em*^ are experimentally measurable “0–0” energy for absorption and emission. Note that within the IMDHO model, the zero-point vibrational energy and the intramolecular reorganization energy are the same for both the |*g*⟩ and |*e*⟩ electronic states.

The vertical absorption (*E*_*v*_^*Abs*^) and emission energy (*E*_*v*_^*Em*^) are obtained directly from TD-DFT for each XCF, and a direct comparison with the experimental *E*_00_^*Abs*^ and *E*_00_^*Em*^ observables requires an additional vibrational calculation (see Figure 4). Alternatively, the measured *E*_00_^*Abs*^ and *E*_00_^*Em*^ can be converted to vertical transition energies (*E*_*v*_^*Abs*^ and *E*_*v*_^*Em*^) if the intramolecular reorganization energy is known, as follows (Figure 4):
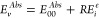
5
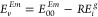
6Where *RE*_*i*_^*g*^ and *RE*_*i*_^*e*^ are the intramolecular energy reorganization for the ground and excited electronic state, respectively. From the theoretical line shape modeling (discussed in the SI), the ground and excited PESs have the same frequencies. In this model, *RE*_*i*_^*g*^ and *RE*_*i*_^*e*^ are the same quantity, calculated as shown in eq 3. *RE*_*i*_ is obtained after fitting the experimental data (Table 4), allowing the conversion of the experimental observables (*E*_00_^*Abs*^ and *E*_00_^*Em*^) into quantities that can be directly compared with TD-DFT outputs (i.e., *E*_*v*_^*Abs*^ and *E*_*v*_^*Em*^). Thus, we explore the performance of the XCFs and TD-DFT to describe excited-state properties influenced by the shape of the excited-state PES and not just their accuracy in reproducing ground state frequencies.

**Figure 5:**
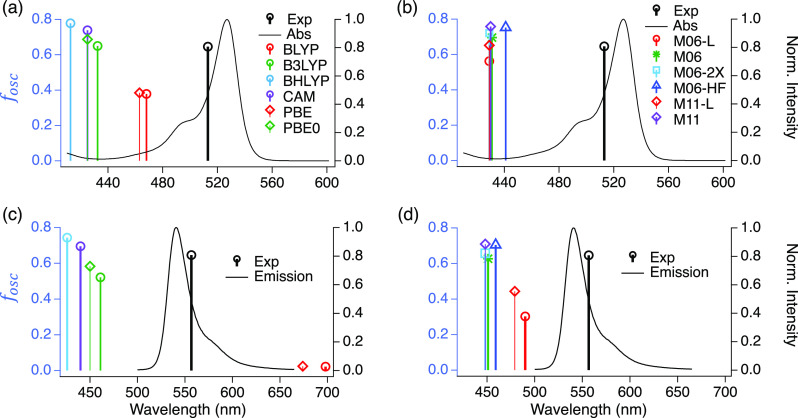
Experimental (black) and TD-DFT-calculated oscillator strengths (*f*_*osc*_^*Abs*^) plotted against the vertical absorption energies (*E*_*v*_^*Abs*^) for each (a) GGA and (b) mGGA XCF. TD-DFT-calculated vertical emission energies (*E*_*v*_^*Em*^) for each (c) GGA and (d) mGGA XCF. The experimental absorption (a,b) and emission (c,d) spectra are included. For parts (c) and (d), we use the same *f*_*osc*_^*Abs*^ as in parts (a) and (b), obtained from integrating the absorption spectra. Here “CAM” corresponds to CAM-B3LYP. Vertical energies were computed at the SS-PCM/TD-DFT level, and *S*_1_ geometry optimization was performed at the LR-PCM/TD-DFT level with benzene as the PCM solvent.

Figure 5(a,b) compares the accuracy of the 12 XCFs to match the experimental *E*_*v*_^*Abs*^ and the oscillator strength (*f*_*osc*_^*Abs*^) associated with the lowest-energy vertical transition (*S*_0_ → *S*_1_) at the ground state optimized geometry. The experimental oscillator strength was obtained by integrating the absorption spectrum in the usual manner,^51^ and the experimental *E*_*v*_^*Abs*^ was determined from eq 5. Additionally, we calculated the *f*_*osc*_^*Abs*^ and the *E*_*v*_^*Abs*^ associated with the first 10 electronic transitions for all 12 XCFs, and we compared them with the experimental absorption spectra from 200 to 600 nm in Figure S3. We observed that local GGAs (BLYP and PBE) identify two equally strong low-energy transitions; conversely, all other XCFs showed one, as observed experimentally. Furthermore, the NTOs corresponding to the *S*_0_ → *S*_1_ transition for BLYP, PBE, and M06-L are drastically different from all others (Figure S4 and Tables S2 and S3). We also notice (Figure S3) an increase in the energy separation between the *S*_0_ → *S*_1_ and *S*_0_ → *S*_2_ transition energies as the HFX amount increases.

From Figure 5(a), it is clear that local GGAs (BLYP and PBE) estimate the lowest *E*_*v*_^*Abs*^ (the closest to the experiment), but their *f*_*osc*_^*Abs*^ is around half the experimental result, and their prediction of two low-energy transitions (Figure S3) is inconsistent with experiments. B3LYP, CAM-B3LYP, and PBE0 all behave similarly to each other; the estimated values of *E*_*v*_^*Abs*^ and *f*_*osc*_^*Abs*^ are close to each other, while BHLYP overestimates both properties the most. In (b), the local mGGAs (M06-L and M11-L) predict the highest *E*_*v*_^*Abs*^ and the lowest *f*_*osc*_^*Abs*^ among all of the mGGAs. M06, M06-2X, M06-HF, and M11 results are close to each other, and M06-HF gives the lowest *E*_*v*_^*Abs*^. Interestingly, we noticed that *f*_*osc*_^*Abs*^ increases with the HFX amount included by the XCF, regardless if they are GGAs or mGGAs.

Figure 5(c,d) shows the experimental and TD-DFT-calculated *E*_*v*_^*Em*^ and *f*_*osc*_^*Abs*^ for the *S*_1_ → *S*_0_ transition at the *S*_1_ optimized geometry. Local GGAs (BLYP and PBE) in panel (c) show the worst performance; they predict an extremely low-energy transition with negligible *f*_*osc*_^*Abs*^, in contrast to the strongly fluorescent characteristics of the dye. Experimentally, the Bodipy has a fluorescent quantum yield of 0.78 in dichloromethane.^74^ As observed in panel (a), panel (c) shows that the largest *f*_*osc*_^*Abs*^ (at the optimized *S*_1_ configuration) corresponds to the XCFs that include the most HFX. In panel (d), we observed that the local mGGAs (M06-L and M11-L) underestimate the *f*_*osc*_^*Abs*^, but the energy is close to that predicted by other GH-mGGAs with different HFX. Here, the *E*_*v*_^*Em*^ predicted by M06, M06-2X, and M11 is almost the same, with a difference in the *f*_*osc*_^*Abs*^ that depends on the amount of HFX; the more HFX, the higher the *f*_*osc*_^*Abs*^.

**Figure 6:**
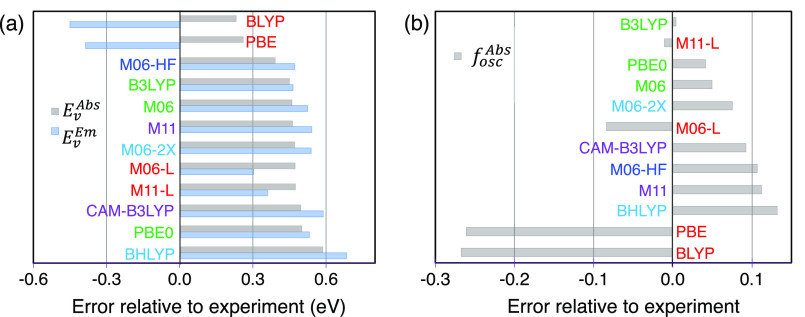
Differences between the DFT-calculated (a) *E*_*v*_^*Abs*^, *E*_*v*_^*Em*^, and (b) *f*_*osc*_^*Abs*^ with the experiment.

Figure 6 shows the error between the experimental and TD-DFT-calculated *E*_*v*_^*Abs*^ and *E*_*v*_^*Em*^ for every XCF, where the error is the algebraic difference between the TD-DFT-calculated property and the experimental value. The bar graphs are sorted from minimum to maximum based on the *E*_*v*_^*Abs*^ error. Despite the other nonphysical predictions, BLYP and PBE perform better than the other XCFs that, regardless of their HFX, had a similar error (∼0.4–0.6 eV) for estimating *E*_*v*_^*Abs*^. On the other hand, the *E*_*v*_^*Em*^ lowest error was obtained with M06-L and M11-L (Figure 6(a)), while the other functionals have similar accuracy (∼0.4–0.6 eV).

Figure 6(b) summarizes the accuracy of the XCFs to calculate the *f*_*osc*_^*Abs*^ observable. In this case, we observe that the best description is achieved by B3LYP (20% HFX), M11-L (0%), PBE0 (25%), and M06 (27%); except for M11-L, the other functionals have a similar HFX contribution but a distinct functional form. The worst performance corresponds to the two local GGA functionals, PBE and BLYP, that drastically underestimate the magnitude of *f*_*osc*_^*Abs*^. Here, we observed a trend where the best performance is achieved by the HFX contribution rather than by being an mGGA or GGA functional. Only local XCFs underestimate *f*_*osc*_^*Abs*^, while the GH and RSH functionals overestimate it proportionally to the HFX.

As mentioned, it is common to determine the performance of XCFs in the excited state by their ability to match the experimental *E*_00_^*Abs*^; therefore, BLYP and PBE might be used to interpret the excited-state properties of Bodipy or similar dyes. However, Figures 5(c,d), S3, and S4 show that these local GGAs results have significant inconsistencies with the experimental observations, and their results differ from those obtained with other GHs and RSHs.

Even though this work aims not to evaluate or compare the impact of the SS versus LR, we observed that all of the *E*_*v*_^*Abs*^ and *E*_*v*_^*Em*^ computed at the SS-PCM/TD-DFT level occurred at higher energy (shorter wavelengths) compared to the LR model, except for the emission in PBE0 and BLYP (Tables S2 and S3). On average, the *E*_*v*_^*Abs*^ and *E*_*v*_^*Em*^ calculated with SS formalism were 965 and 995 cm^–1^ higher than the results obtained from LR (not considering *E*_*v*_^*Em*^ from PBE0 and BLYP). Because of this, we would get a lower error using the vertical energies calculated at the LR-PCM/TD-DFT level; however, LR is not an appropriate method for including solvent effects during the emission process because the exact excited-state electron density is never computed, and the ground state is always in equilibrium with the solvent degrees of freedom.^73^ These deficiencies are not present in the SS model. Thus, we consider SS a more robust approach, and it was used for calculating vertical energies (*E*_*v*_^*Abs*^ and *E*_*v*_^*Em*^) throughout this work.

### Mapping the Excited-State PES

We propose benchmarking the performance by the XCFs’ ability to describe the excited-state PES rather than just the electronic transition’s strength and energy. Here, we can discount the error related to calculating vertical energies (*E*_*v*_^*Abs*^ and *E*_*v*_^*Em*^); instead, we use the energy difference associated with the relaxation at the excited-state PES, the *RE*_*T*_^*DFT*^ (eq 4). The former requires an excited-state geometry optimization that includes the solvent. Then, the result can be directly compared with the experimental description of the excited-state PES (within the standard approximations) obtained from the simultaneous fitting of the RR profiles of all modes and absorption and emission spectra, *RE*_*T*_ (eq 3). It is worth pointing out that all TD-DFT calculations included the solvent in the same fashion: *S*_1_ geometry optimization was performed at the LR-PCM/TD-DFT level, and *E*_*v*_^*Em*^ and *E*_*v*_^*Abs*^ were computed at the SS-PCM level. This means that the error in estimating the experimental *RE*_*T*_^*DFT*^ value is attributed to the accuracy of each XCF in describing the excited-state PES and its electronic density.

**Figure 7:**
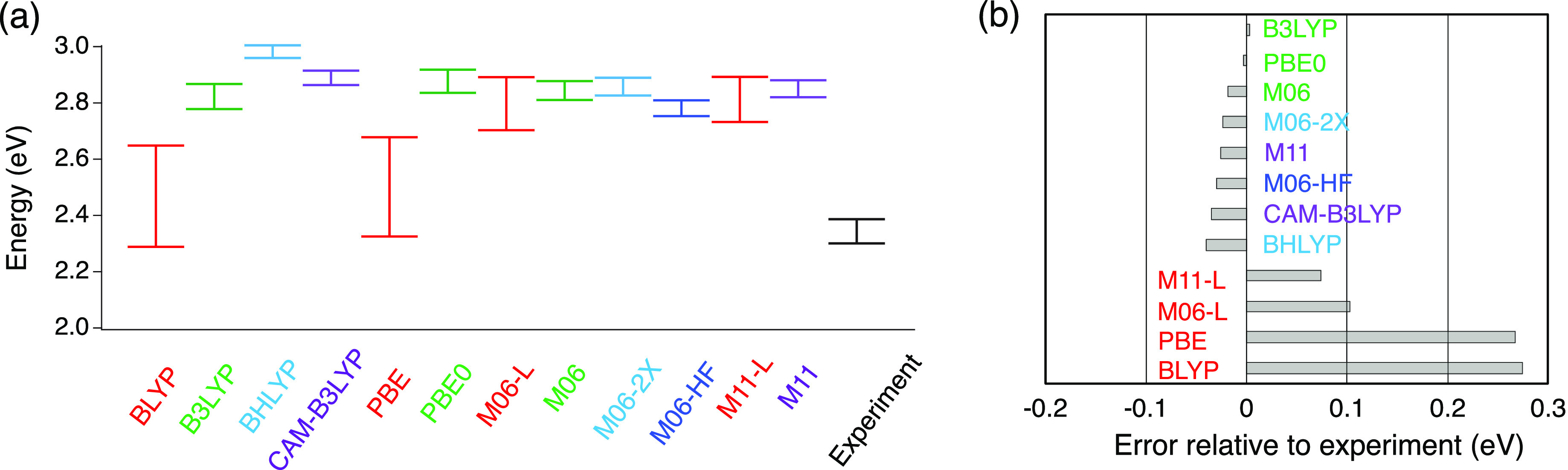
(a) Each bar shows the *RE*_*T*_^*DFT*^ for every XCF, where the upper bar is *S*_1_(*S*_0_) and the lower bar is given by *S*_1_(*S*_1_). The experimental *RE*_*T*_ (black) is given by *E*_*v*_^*Abs*^ and (*E*_*v*_^*Abs*^ – *RE*_*T*_). (b) The error is calculated as (*RE*_*T*_^*DFT*^ – *RE*_*T*_). Vertical energies were computed at the SS-PCM/TD-DFT level, and *S*_1_ geometry optimization was performed at the LR-PCM/TD-DFT level, with benzene as the PCM solvent.

Figure 7(a) presents the *RE*_*T*_^*DFT*^ for each XCF. The upper bar corresponds to the energy of *S*_1_(*S*_0_), and the lower bar corresponds to the energy of *S*_1_(*S*_1_), and the difference between these energies is *RE*_*T*_^*DFT*^. The experimental value (black) is given by *E*_*v*_^*Abs*^ and the total reorganization energy (eq 3), i.e., the upper bar is *E*_*v*_^*Abs*^, and the lower bar is *E*_*v*_^*Abs*^ – *RE*_*T*_. In panel (b), we present the error associated with the *RE*_*T*_^*DFT*^ for each XCF and experimental *RE*_*T*_, calculated as the algebraic difference. We use this criterion to determine which XCF better describes the excited-state PES. As before, each XCF is labeled according to the amount of HFX included.

Figure 7(a,b) shows the relevance of HFX to describe the excited-state PES accurately. Even though BLYP (0%), B3LYP (20%), BHLYP (50%), and CAM-B3LYP (19–65%) have similar functional forms, their performance dramatically depends on the HFX included. The best result is obtained with B3LYP, followed by CAM-B3LYP, BHLYP, and BLYP. Ignoring HFX leads to overestimating the *RE*_*T*_, while including too much HFX leads to underestimating the energy difference between *S*_1_(*S*_1_) and *S*_1_(*S*_0_). A similar dependence was observed with the *f*_*osc*_^*Abs*^, where too much HFX led to overestimating its magnitude and not including HFX led to the opposite.

We observed the same situation for the M06 family: M06-L (0%), M06 (27%), M06-2X (54%), and M06-HF (100%). The best agreement with the experiment is observed with M06, followed by M06-2X, M06-HF, and M06-L. Similarly, not including HFX leads to overestimating *RE*_*T*_^*DFT*^, while too much HFX leads to the opposite. The M11 and PBE families show the same trend.

Comparing *RE*_*T*_ magnitudes represents a comparison of the total reorganization, including all normal modes in the system, as well as the dielectric response of the solvent. The percentage of HFX is critical to match experimental observations rather than the type of functional, GGA, or mGGA. Interestingly, a higher contribution of HFX does not lead to better performance, but not including HFX at all leads to the worst results (regardless of the functional) as observed for BLYP, PBE, M06-L, and M11-L.

In order to obtain a more precise understanding of the shape of the DFT-predicted PES, it is necessary to explore the separate contributions of the *RE*_*T*_ from individual vibrational modes. This can be done by comparing the RREPs.

### Resonance Raman Excitation Profiles

The RR excitation profile is calculated for every XCF studied in this work. The profiles require the FC displacements for all modes ({Δ_*k*_}), the ground state frequencies (both calculated from DFT/TD-DFT), and the molecular parameters from the fitting process (Table 3). Then, using eqs S3–S5, we generate the absorption, emission, and RREP for each XCF, allowing for a direct comparison with the experiment. Here, we chose four peaks (241, 602, 1172, and 1410 cm^–1^), representing different spectral regions, to evaluate the performance of different XCFs.

**Figure 8:**
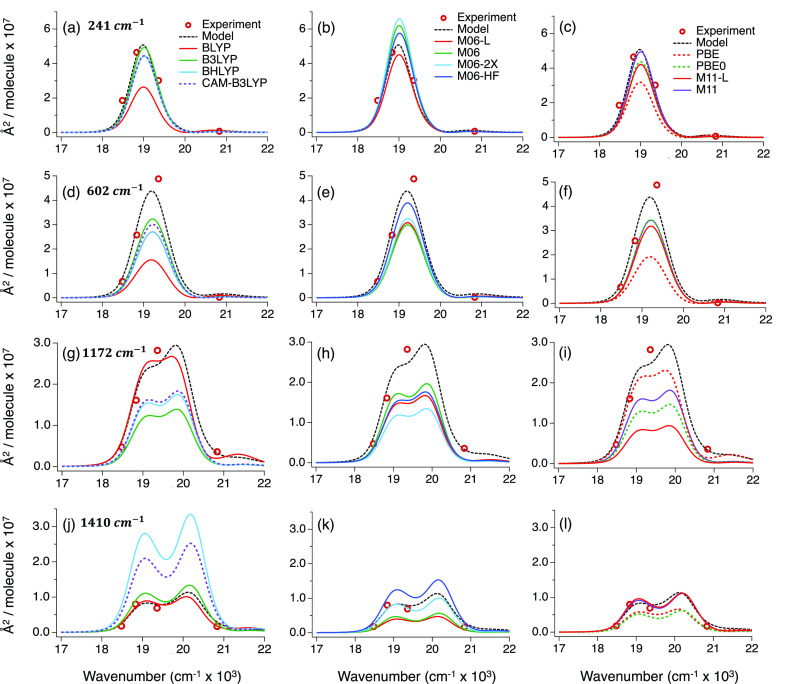
Comparison between the TD-DFT/DFT-calculated, experimental (points), and modeled (black dashed) RR excitation profile for four vibrational modes: 241 (a–c), 602 (d–f), 1172 (g–i), and 1410 (j–l) cm^–1^. On top of the experimental and modeled profiles are the DFT profiles calculated using the labeled functionals.

Figure 8 presents the RR excitation profile for each functional. The column on the left shows the results for BLYP, B3LYP, BHLYP, and CAM-B3LYP; the middle column shows the results obtained from M06-L, M06, M06-2X, and M06-HF; and the right column shows the results from PBE, PBE0, M11-L, and M11. The experimental cross section (circles) and the experimentally modeled RREP (black dashed in Figure 8, equivalent to the black line in Figure 3c–e) are also included. Figure 8 allows one to access how well an XCF predicts the excited-state PES projected onto individual normal modes of the molecule. An overestimate of the Raman cross section indicates that XCF predicts too large a force along that normal mode in the FC region of the *S*_1_ surface compared to the experiment.

**Figure 9:**
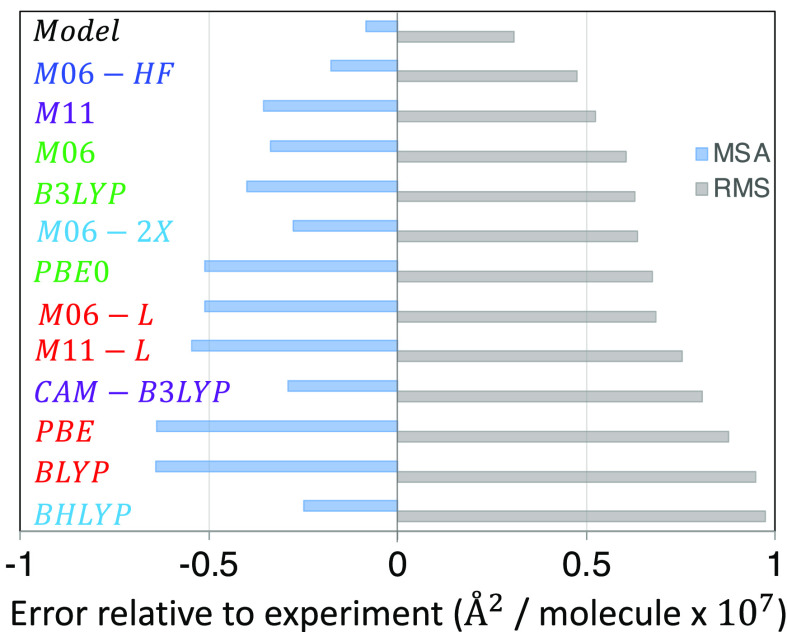
RMS and MSA difference (in Å^2^/molecule) between the experimental and calculated RREP. “Model” is the IMDHO fitting to the experimental data; DFT results are ranked from the best to worst.

To quantify the accuracy of different XCFs to match the experimental RREP (circles), we calculate the mean signed average (MSA) and the root-mean-square (RMS) between the calculated RREP and the experimental data points. This is performed for each of the four modes considered here (Figure S5), and then, the four modes’ results are averaged. Figure 9 shows the overall performance for each XCF. M06-HF and M11, the XCFs that include the largest percentages of HFX, achieved the best performance in this test. They are followed by M06 and B3LYP, and M06-2X, with almost identical RMS value. The worst performance was observed for the four local XCFs (BLYP, PBE, M06-L, and M11-L), CAM-B3LYP, and BHLYP. The negative MSA for all cases shows that the forces along the normal modes, on average, are being underestimated by the XCFs used and TD-DFT, compared to those in the experiment.

The analysis of this section partially agrees with the results in Figure 7. The first six XCFs with the best performance are the same in both analyses. The difference arises in their ordering; while considering *RE*_*T*_, the XCFs with an HFX between 20 and 30% are the best, but for the RREPs, the best performance is observed for those with the highest HFX. Although we only used four modes in this analysis instead of the 20 modes identified with RR, we highlight the Raman cross section’s explicit dependence on all frequencies and FC displacements (eqs S3–S6). Hence, matching the experimental RREP is an indication of an accurate description of the multidimensional excited-state PES in the FC region.

The four modes considered in this section were chosen because they are easily identified and provide a general picture of different regions of the RR spectra. The assignment of the experimental modes with DFT is based on the frequency alignment and the magnitude of the FC displacement (Δ). However, due to the spectral resolution (∼20 cm^–1^) and the spectral density of vibrational modes identified with DFT, a complete assignment of each experimental peak with DFT is extremely challenging and prone to error. In the next section, we present a strategy that includes all modes of the RR spectra at different excitation wavelengths.

### Benchmarking XCFs Using RR Spectra

Here, we compare the RR spectra obtained from different XCFs and the experimental RR spectra collected using FSRS at different excitation wavelengths: 541, 530, and 516 nm. The RR spectrum collected at 480 nm is not considered here, since it is relatively weak and provides no information on the low-frequency region (Figures S1, S2, and 3). The RR spectra at a specific excitation wavelength can be constructed from the RREP described in the previous section. By including the vibrational damping factor chosen so that the Raman bands have widths similar to those observed experimentally (∼20 cm^–1^), we can directly compare the experimental and the DFT/TD-DFT-calculated RR spectra at different excitation wavelengths.

**Figure 10:**
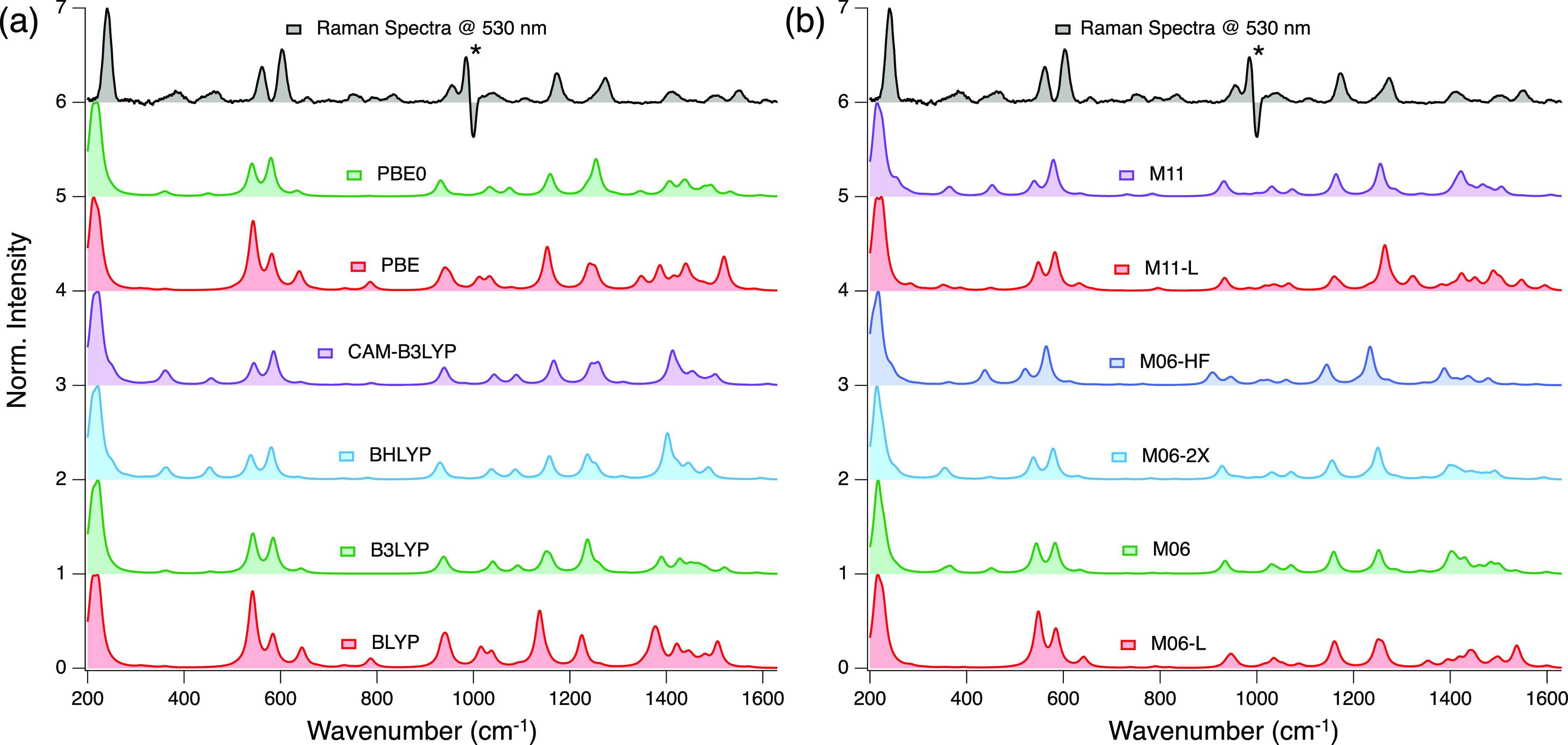
Experimental (black) and DFT-calculated RR spectra using (a) GGA and (b) mGGA XCFs. The excitation wavelength is 530 nm. All of the RR spectra are normalized relative to the most intense peak.

Figure 10 presents the experimental RR spectra at 530 nm and the DFT-computed RR spectra for the (a) GGA (BLYP, B3LYP, BHLYP, CAM-B3LYP, PBE, and PBE0) and (b) mGGA (M06-L, M06, M06-2X, M06-HF, M11-L, and M11) XCFs considered in this work. The normal-mode frequencies for all XCFs have been scaled according to the literature scaling factors, presented in Table 1, and we use the same color labeling as in previous figures. The experimental and DFT-computed RR spectra at 541 and 516 nm can be found in SI (Figures S6 and S7).

In (a), we found that the ability of XCFs to reproduce the experimental RR spectra in the low-frequency region depends on the percentage of HFX. Local XCFs (BLYP and PBE) fail to show the features observed at 382 and 462 cm^–1^ and overestimate the intensity of the 653 cm^–1^ peak. B3LYP and PBE0 barely identify the 382 and 462 cm^–1^ peaks, but the 653 cm^–1^ intensity is closer to that of the experiment. Finally, BHLYP and CAM-B3LYP correctly identify the peaks mentioned, and their relative intensities are closer to the experimental RR spectra. PBE0 and B3LYP do better in the high-frequency region (>1000cm^–1^) and do not overestimate the intensity of peaks in this part of the spectrum, as BHLYP, CAM-B3LYP, PBE, and BLYP do.

A similar scenario is observed in (b). Local XCFs (M06-L and M11-L) cannot reproduce the low-frequency regions’ 382 and 462 cm^–1^ peaks. XCFs with higher HFX do better in this region, notably M11 and M06. In the high-frequency region, the calculated spectra obtained with M06-2X and M06-HF show a closer resemblance with the experiment. From these qualitative observations, we claim that BHLYP, CAM-B3LYP, M06, and M11 are better XCFs to study the low-frequency region, and PBE0, B3LYP, M06-2X, and M06-HF are better for the high-frequency part of the spectrum.

To determine, quantitatively, which XCF describes better the experimental RR spectra, we define the overlap factor (*OF*) as
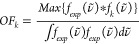
7

Here,  is the experimental RR spectra,  is the DFT-calculated RR spectra using the XCF *k*, and *k* is an index that runs over all XCFs used in this work, shown in Table 1. The numerator is a cross-correlation of the experimental and TD-DFT spectra and, at its maximum, expresses the amount of overlap when  is shifted over . In Figure S8, we plot  and  when the overlap between the experimental and TD-DFT-calculated RR spectra is maximum. The denominator corresponds to the maximum overlap possible, ensuring that the *OF* is a number between 0 and 1, where 1 corresponds to a perfect overlap between the experimental and DFT-calculated RR spectra. All experimental and DFT-calculated RR spectra are normalized before calculation of the *OF*, as follows:
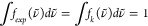
8

**Figure 11:**
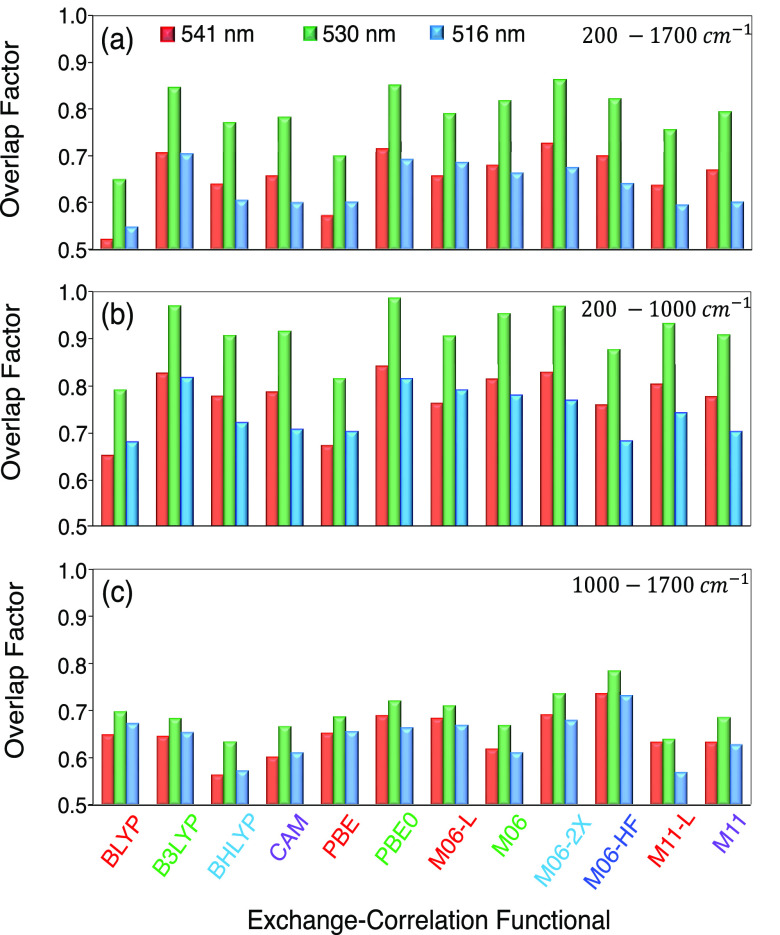
(a) Overlap factor obtained at three different excitation wavelengths: 541 (red), 530 (green), and 516 (blue) nm. The overlap factor was also calculated for the (b) low-frequency (200–1000 cm^–1^) and (c) high-frequency (1000–1700 cm^–1^) regions of the RR spectrum. Here, “CAM” corresponds to CAM-B3LYP.

In this way, we hope to test the ability of each XCF to produce an RR spectrum that matches the frequencies and relative intensities of the experimental spectrum. Figure 11(a) shows the *OF* for each XCF at 541, 530, and 516 nm. Overall, the best performance is obtained with M06-2X, PBE0, and B3LYP, closely followed by M06 and M06-HF. Conversely, M11-L, PBE, and BLYP show the worst performance. Interestingly, if we compare Figures 7(b), 9, and 11(a), sorted from the best to worst performance, we observe that the first six XCFs are the same. At 516 nm, the OFs followed a similar trend; however, there are some significant differences. M06-L behaves as well as B3LYP and PBE0, and M11 performs as badly as CAM-B3LYP, PBE, and BLYP.

Figure 11 also presents the *OF* in the low- and high-frequency regions of the spectrum, (b) between 200 and 1000 cm^–1^ and (c) 1000–1700 cm^–1^. On average, the *OFs* are 0.78, 0.91, and 0.74 in the low-frequency region for the 541, 530, and 516 nm excitation wavelengths, respectively. These values decreased in the high-frequency region to 0.65, 0.69, and 0.64. The former shows that the XCFs considered here can better reproduce the RR spectrum in the low-frequency region for the Bodipy molecule.

There are advantages and disadvantages of this *OF* comparison. Though it is possible to quantitatively assess the accuracy of XCFs to reproduce the full experimental RR spectrum, the units of the RR intensity are arbitrary, because the normalization process affects the absolute intensities (eq 8). Furthermore, the frequency scaling factor, presented in Table 1, significantly impacts the alignment of the experimental and DFT Raman peaks and thus the *OF*. For instance, the *OF* for B3LYP and BHLYP at 541 nm using the scaling factor presented in Table 1 is 0.692 and 0.628, respectively; however, if we do not scale the frequency (scaling factor = 1), the *OF* is 0.599 and 0.551. Clearly, changes to the scaling factor will affect the alignment of the theoretical and experimental spectra, so *ad hoc* adjustments based on a single molecule’s alignment could significantly improve the *OF*.

Among the GGAs, PBE0 and B3LYP show the highest *OF*; however, these XCFs could not correctly capture the features observed at 382 and 462 cm^–1^. Conversely, CAM-B3LYP and BHLYP reproduce the peaks mentioned, but their *OF* is lower. For the mGGAs, only M06 and M11 capture the 382 and 462 cm^–1^ peaks, unlike M06-2X XCF, which has the highest *OF*. CAM-B3LYP, BHLYP, M06, and M11 are the XCFs that correctly identify the peaks in the 300 to 700 cm^–1^ region observed experimentally, and through the *OF*, we can determine which of these XCFs properly captures the relative height and position of the overall Raman spectrum. From Figure 11, it is clear that M06 is slightly better in the low- and high-frequency regions and at different excitation wavelengths and that M11, CAM-B3LYP, and BHLYP have a similar performance.

In the analysis of the RREP (previous section), M11, M06-HF, and M06 showed the best performance (Figure 9), while CAM-B3LYP and BHLYP were among the worst, comparable to the local XCFs. We conclude that M06 and M11 are the XCFs that best describe the excited-state PES of the Bodipy. It is worth pointing out that M06-HF showed a remarkable performance in the high-frequency region, spanning from 1000 to 1700. Additionally, M06-HF estimates the RREP with the least error (Figure 8).

## Conclusions

We extensively studied the performance of different XCFs and TD-DFT to describe the excited state of Bodipy. The computational results obtained with DFT/TD-DFT can be directly compared with those of their experimental counterparts. This is achieved using theoretical optical line shape functions that include the Brownian oscillator model to fit experimental RR excitation profiles, absorption, and emission spectra, providing detailed information about the molecule upon photoexcitation and further relaxation.

First, we used traditional strategies to benchmark XCFs and TD-DFT. We compared vertical excitation energies (*E*_*v*_^*Abs*^ and *E*_*v*_^*Em*^) and *f*_*osc*_^*Abs*^ obtained from TD-DFT, with the experimental vertical transitions (eqs 5 and 6) and oscillator strength determined from the absorption spectrum and the optical line shape analysis. We observed that considering only the energy difference between experimental and calculated *E*_*v*_^*Abs*^ as the main criterion to benchmark the performance of XCFs in the excited state can be deceiving. This criterion incorrectly suggests that the local functionals have outstanding performance; however, further analysis showed their inability to match experimental observation. In particular, local GGAs predict that the absorption from the ground electronic state occurs to two equally bright states and that, at the *S*_1_ minimum configuration, the *S*_1_ → *S*_0_ transition has a negligible oscillator strength (dark) in a highly fluorescent molecule such as Bodipy. Moreover, the results obtained by comparing vertical transition and oscillator strength are inconsistent, motivating further analysis of the XCF predictions of the *S*_1_ PES and the search for a better and more robust criterion to judge the accuracy of different XCFs in the excited state.

Second, we mapped the excited-state PES by calculating the total reorganization energy, *RE*_*T*_^*DFT*^. A direct comparison with the experimental *RE*_*T*_ allows us to assess the performance of different XCFs and TD-DFT to describe the excited-state PES. This criterion is more robust than the previous energy difference and requires no ground-state calculations. As a result, we observed a clear trend where all XCFs that include between 20 and 30% of HFX (B3LYP, PBE0, and M06) performed the best, regardless of their functional form. We noticed that XCFs with no HFX overestimate the *RE*_*T*_, while XCFs with the largest amount of HFX underestimate it.

Third, we compute the RREP for four modes (241, 602, 1172, and 1410 cm^–1^), representing different spectral regions, and compare it with the experimental profile. As observed before, the worst performance corresponds to the local functionals (BLYP, PBE, M06-L, and M11-L), BHLYP, and CAM-B3LYP. Conversely, the best performance was achieved by the XCFs with the highest HFX (M06-HF and M11), followed by M06, B3LYP, M06-2X, and PBE0. Due to the difficulty of assigning Raman peaks, we could not include all of the peaks identified with RR, limiting the number of modes considered. However, we consider this a robust criterion that explicitly includes all FC displacements, frequencies, and molecular parameters shown in Table 3. Agreement with the experimental cross sections indicates an accurate description of the multidimensional energy gradient at the FC region, a term that determines the dynamics.

Fourth, we proposed a new overlap factor, corresponding to the maximum in the cross-correlation of the normalized DFT and experimental RR spectra, to evaluate the accuracy of each XCF to reproduce the shape of the experimental RR spectra at different excitation wavelengths. This complementary analysis confirms the poor performance of local functionals (BLYP, PBE, M06-L, and M11-L), CAM-B3LYP, and BHLYP. Similarly, it confirms the superiority of B3LYP, PBE0, M06, M06-2X, M06-HF, and M11 describing Bodipy’s excited-state properties.

Throughout this work, we have proposed different criteria to benchmark the performance of XCFs in describing the electronic excited-state properties beyond the simple comparison of vertical excitation energies. By evaluating the ability of the XCFs to describe the *RE*_*T*_ and the forces at the FC region, we showed the crucial importance of HFX for correctly describing the excited-state PES, being more relevant than the functional form. Our results consistently showed the XCFs that accurately describe the Bodipy’s excited-state PES (B3LYP, PBE0, M06, M06-2X, M06-HF, and M11) and those that should be avoided (BLYP, PBE, M06-L, M11-L, BHLYP, and CAM-B3LYP). Finally, we believe that the XCFs that best describe Bodipy’s excited-state PES are M06 and M11.

The simultaneous fitting of the absorption, fluorescence, and all RREPs is an extensive task that requires collecting FSRS spectra at different excitation wavelengths and adjusting *N* + 4 variables (*N* Δ’*s* for *N* vibrational modes, *E*_0_, Γ, θ, and μ). The oscillator strength is a more robust criterion than the vertical excitation energies, and it is closer to the results obtained with a more extensive analysis employed here (Figures 6, 7, 9, and 11). Further, by calculating the *f*_*osc*_ and *E*_*v*_^*Abs*^ of the first 10 electronic transitions (Figure S3), we easily identified those XCFs that could not reproduce well-known properties observed by simple steady-state methods (Figure S3 and Tables S2 and S3). The oscillator strength can be easily obtained from both the experimental absorption spectra and TD-DFT. We encourage the use of *f*_*osc*_^*Abs*^ over *E*_*v*_^*Abs*^ as a tool to choose the correct XCFs for a particular molecular system whenever a more sophisticated methodology is not possible.

The methodology presented here can be applied to any other molecule that absorbs in the visible range, and the conclusion drawn in this work can be applied for the Bodipy or molecules with similar structure.

## Supporting Information Available

Description of the theoretical line shape functions used in this work; raw and baseline subtracted FSRS spectra and resonance Raman cross section at each wavelength; TD-DFT transition properties; additional information for calculating RMS, MSA, and the overlap factor (PDF)
