# Supplementary material for: The Best Models of Bodipy’s Electronic Excited State: Comparing Predictions from Various DFT Functionals with Measurements from Femtosecond Stimulated Raman Spectroscopy
Source: J Phys Chem A. 2023 Sep 26;127(39):8238–51. doi: 10.1021/acs.jpca.3c05040 (PMC10561280; doi:10.1021/acs.jpca.3c05040)
Supplement: Supplementary file 1 — jp3c05040_si_001.pdf [file jp3c05040_si_001.pdf]

Supporting Information for:

The Best Models of Bodipy's Electronic Excited State: Comparing Predictions from Various DFT Functionals with Measurements from Femtosecond Stimulated Raman Spectroscopy

Juan S. Sandoval† and David W. McCamant. †

†Department of Chemistry, University of Rochester, New York 14627, United States.

Table of Contents

Theoretical optical line shape analysis 2
Raw FSRS data with baseline 5
FSRS Spectra 6
Resonance Raman Cross-Section 7
Oscillator Strength and Vertical Transition Energies 8
TD-DFT Electronic Transition Properties 9
Natural Transition Orbitals 11
RMS and MSA for each mode considered 12
Additional Resonance Raman Spectra 13
Maximum overlap between the DFT calculated and experimental RR spectra at 530 nm. 14
Bibliography 15

Theoretical optical line shape analysis

Here we summarize the optical line shape functions implemented to extract molecular information from experiments, a thoroughly discussion of time domain-methods for computing RR line shapes can be found elsewhere [1] [2] [3]. The complete description of the code implemented can be found in Reference [4].

Following the Born-Oppenheimer approximation, the linear coupling model assumes that the ground electronic state ($|\mathrm{g}\rangle$) potential energy surface is described as a set of N independent harmonic modes where each mode k has an associated frequency ω_k.The Hamiltonian for the ground state is:

$$\hat{h}^g = \sum_k^N \frac{\hbar\omega_k}{2}(\hat{p}_k^2 + \hat{q}_k^2) = \sum_k^N \hbar\omega_k\left(\hat{a}_k^\dagger \hat{a}_k + \frac{1}{2}\right) \qquad (S1)$$

Where $\hat{a}_k^\dagger = (\hat{q}_k - i\,\hat{p}_k)/\sqrt{2}$ and $\hat{a}_k = (\hat{q}_k + i\,\hat{p}_k)/\sqrt{2}$ are the usual creation and annihilation operators, respectively. The excited electronic state ($|e\rangle$) potential energy surface, as well, is described as N independent harmonic modes, and each mode has the same ground state frequency ω_k and is displaced by Δ_k from the ground state equilibrium configuration. The Hamiltonian for the excited state is then:

$$\hat{h}^e = \sum_k^N \frac{\hbar\omega_k}{2}[\hat{p}_k^2 + (\hat{q}_k - \Delta_k)^2] = \hat{h}^g - \sum_k^N \left[\frac{\hbar\omega_k\Delta_k}{\sqrt{2}}(\hat{a}_k^\dagger + \hat{a}_k) - \frac{\hbar\omega_k\Delta^2}{2}\right] \qquad (S2)$$

Additionally, within the Condon approximation and assuming only one electronic transition is resonant, the absorption and resonance Raman cross sections (within the first-order approximation) can be computed as follows:

$$\sigma_A(\omega) = \frac{4\pi e^2\omega}{3cn\hbar}\int d\delta\, G(\delta)\, Im\left\{i\int_0^\infty dt\, e^{i(\omega-E_0-\delta)t} D(t)\, A(t)\right\} \qquad (S3)$$

$$\sigma_F(\omega) = \frac{4\pi ne^2\omega}{3c\hbar}\int d\delta\, G(\delta)\, Im\left\{i\int_0^\infty dt\, e^{i(\omega-E_0-\delta)t} D^*(t)\, A^*(t)\right\} \qquad (S4)$$

$$\sigma_R^{(1)}(\omega,\{Q\}) = \frac{8\pi e^4 \omega_S^3 \omega}{9c^4 \hbar^2} \int d\delta\, G(\delta) \left| \int_0^\infty dt\, e^{i(\omega - E_0 - \delta)t} \right.$$

$$\times\ D(t)A(t) \prod_{\alpha(Q_\alpha>0)} \left[\frac{1}{Q_\alpha!}\right]^{\frac{1}{2}} \left(\frac{\pm[1+\bar{n}_\alpha]^{\frac{1}{2}}\Delta_\alpha}{\sqrt{2}}\right)^{Q_\alpha} \left(1 - e^{-i\omega_\alpha t}\right)^{Q_\alpha}$$

$$\times \left. \prod_{\alpha(Q_\alpha<0)} \left[\frac{1}{-Q_\alpha!}\right]^{\frac{1}{2}} \left(\frac{\pm\bar{n}_\alpha^{\frac{1}{2}}\Delta_\alpha}{\sqrt{2}}\right)^{-Q_\alpha} \left(1 - e^{i\omega_\alpha t}\right)^{-Q_\alpha} \right|^2 \qquad (S5)$$

Here E_0 is the energy gap between the zero vibrational levels of $|g\rangle$ and $|e\rangle$ usually calculated as the energy of intersection between the normalized absorption and emission spectra, ω_s is the frequency of the scattered photon, n is the index of refraction, $\bar{n}_k$ is the average thermal occupation number for mode k, Q_k is the change in the vibrational quantum number associated with mode k, and $A(t)$ is the absorption time-correlator given by:

$$A(t) = |M|^2\, exp\left(-\sum_k^N \left[S_k(1+\bar{n}_k)\left(1 - e^{-i\omega_k t}\right) + \bar{n}_k S_k\left(1 - e^{i\omega_k t}\right)\right]\right) \qquad (S6)$$

where $S_k = \Delta_k/2$, the Huang-Rhys parameter. $G(\delta)$ is the static inhomogeneous distribution of E_0 energies that accounts for bath-induced energy fluctuations occurring on timescales slower than the vibrational dephasing, assumed to be a gaussian distribution defined as:

$$G(\delta) = \frac{1}{\theta\sqrt{2\pi}} e^{-\frac{\delta^2}{2\theta^2}} \qquad (S7)$$

Where θ is the static inhomogeneous parameter. $D(t)$ is the lineshape function describing the electronic dephasing, which considers the finite lifetime of electronic coherence, and it is chosen to model the solvent-solute interactions following the Brownian oscillator model in the high-temperature limit and strongly overdamped solvent motion, giving:

$$D(t) = e^{-g(t)} \qquad (S8)$$

Where

$$g(t) = \left(\frac{D}{\Lambda}\right)^2 (\Lambda t - 1 + e^{-\Lambda t}) + i\left(\frac{D^2}{2k_B T\Lambda}\right)(1 - e^{-\Lambda t}) \qquad (S9)$$

Here, Λ is the modulation frequency, D is the solvent coupling strength, K_B is the Boltzmann constant, and $\kappa \equiv \frac{\Lambda}{D}$ is the solvent parameter which is related to the full-width half maxima of the line shape, Γ, an adjustable parameter during the fitting process.

Raw FSRS data with baseline

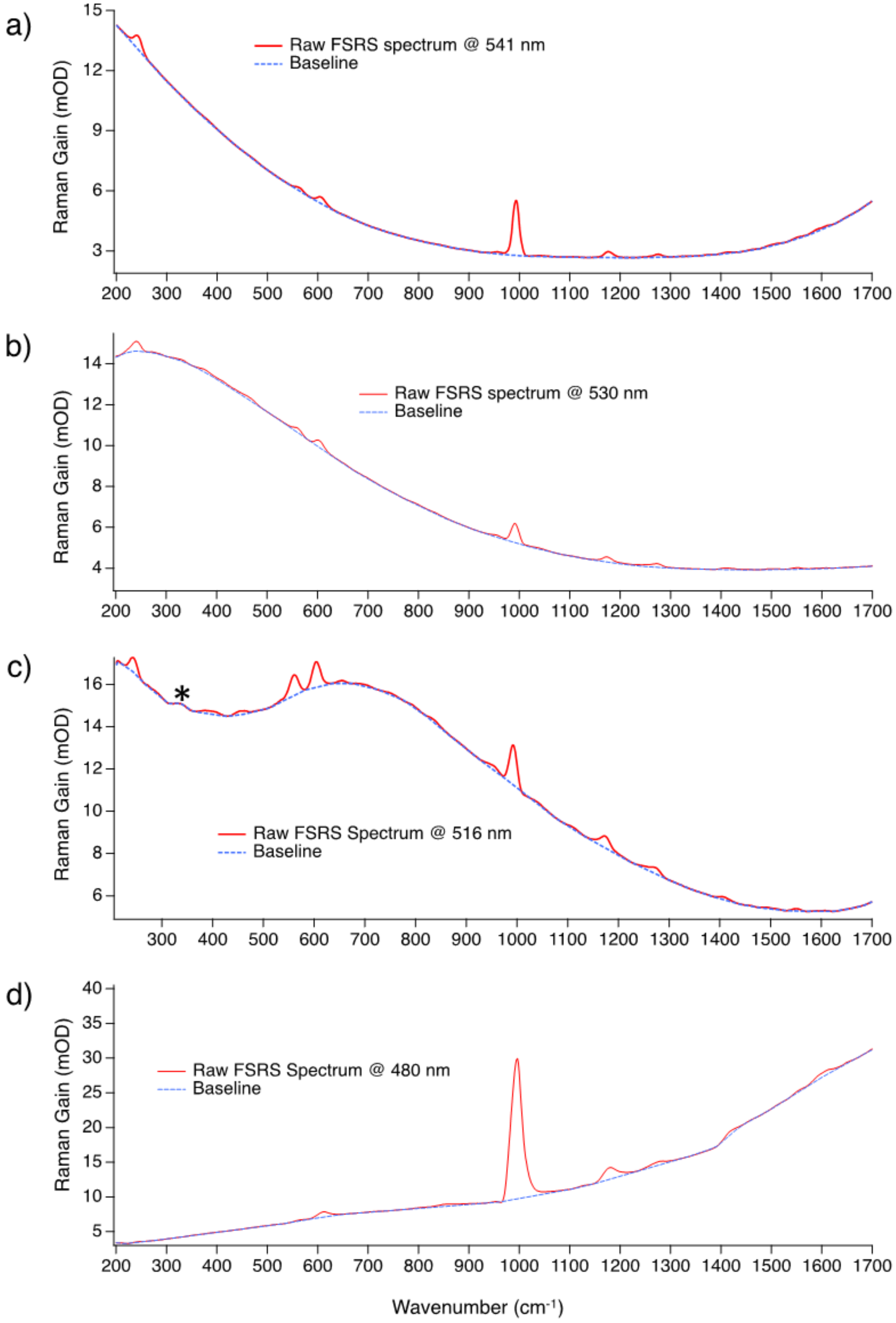

Figure S1. Raw FSRS spectra (red) with baseline drawing (blue) at four different Raman pumps: (a) 541 nm, (b) 530 nm, (c) 516 nm, and (d) 480 nm. The shifted excitation Raman difference spectroscopy (SERDS) showed that the signal marked in (c) with an asterisk is insensitive to small shifts in the Raman pump wavelength.

FSRS Spectra

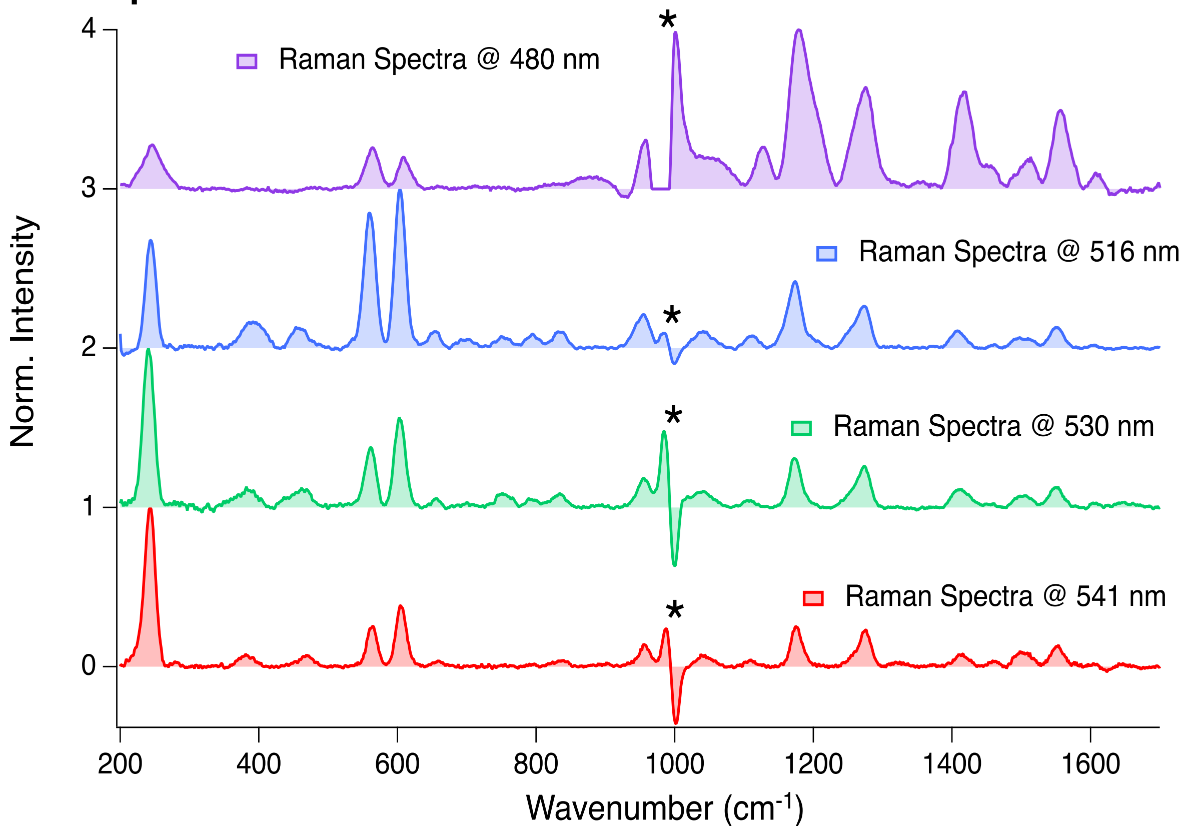

Figure S2. FSRS spectrum after baseline subtraction. All the RR spectra are normalized relative to the most intense peak. The asterisk marks a residual feature from solvent subtraction.

Resonance Raman Cross-Section

Table S1. Bodipy Resonance Raman cross-section for every mode at four different excitation wavelengths. [(a)]

Frequency (cm^{-1})	Raman Cross section ($Å^2$/molecule x 10^7) at different Raman pump wavelenghts (nm)			
	541	531	516	480
241	1.9	4.7	3.0	0.1
383	0	0	1.0	0.0
462	0.2	1.0	1.0	0.0
560	0.4	1.8	4.6	0.0
602	0.7	2.6	4.9	0.0
653	0.1	0.2	0.6	0.0
699	0	0	0.4	0.0
752	0	0.6	0.5	0.0
795	0	0.3	0.5	0.0
834	0.1	0.5	0.6	0.0
955	0.3	1.2	1.5	0.0
1041	0.2	0.8	0.8	0.0
1111	0.1	0.2	0.5	0.0
1172	0.5	1.6	2.8	0.4
1255	0.1	0.8	0.7	0.0
1273	0.4	0.9	1.3	0.2
1410	0.2	0.8	0.7	0.2
1458	0.05	0.13	0.09	0.0
1502	0.3	0.5	0.6	0.0
1551	0.3	0.7	0.7	0.1

[(a)] Cross-sections are obtained with parallel polarization of pump and probe pulses. Depolarization ratio was set to 0.33. Cross-sections calculated by comparison to the differential cross-section of the benzene 992 cm^{-1} mode.

Oscillator Strength and Vertical Transition Energies

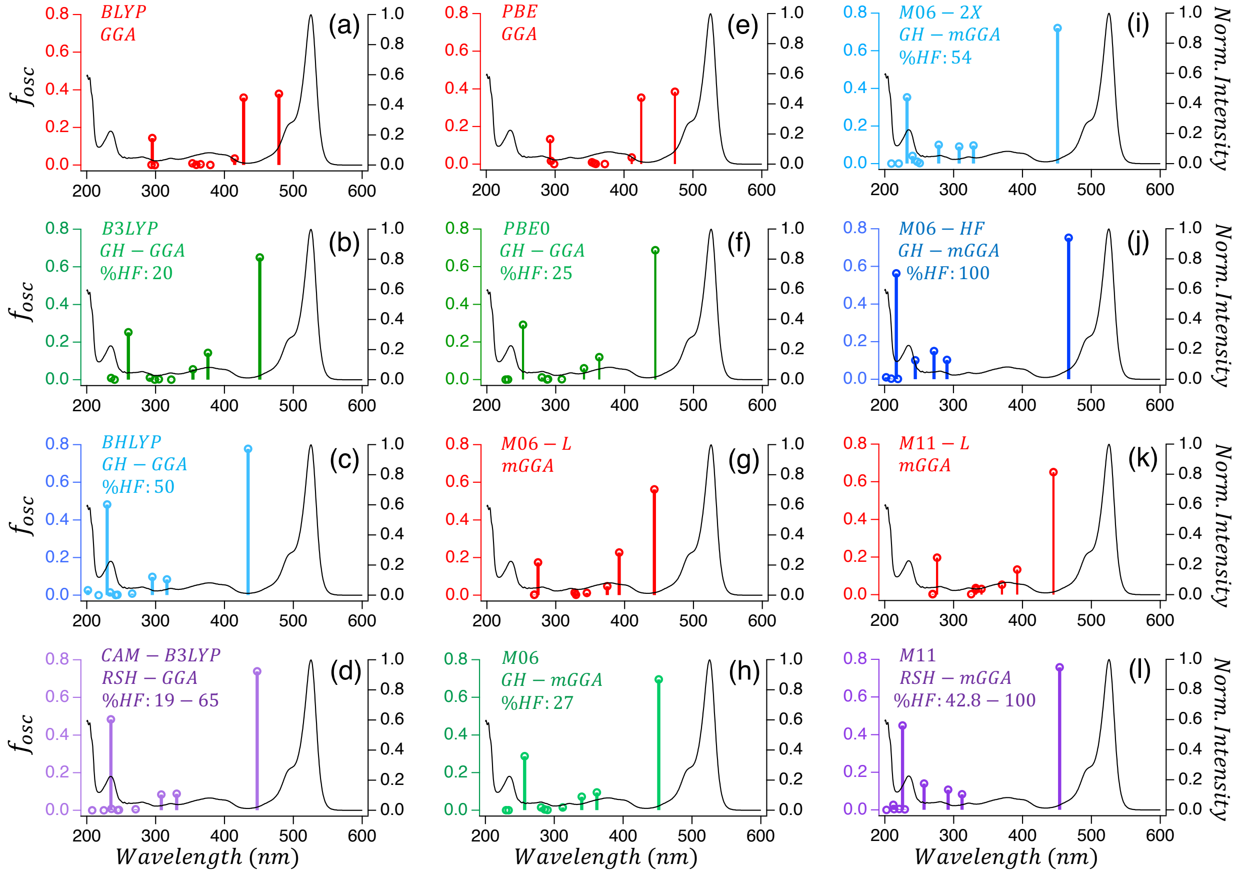

Figure S3. Oscillator strength and vertical excitation energies for the first 10 electronic transitions ($S_0 \rightarrow S_n$, where n=1, 2, 3, 4, 5, 6, 7, 8, 9, 10) for each label XCF. Vertical energies were calculated at the LR-PCM/TD-DFT level. The normalized absorption spectrum for the Bodipy in cyclohexane is included. Here we use cyclohexane, not benzene, to access the region between 200-300 nm and compare it with the TD-DFT output.

TD-DFT Electronic Transition Properties

Table S2. Bodipy lowest energy transitions calculated with the labeled functional in benzene using the ground state optimized geometry. Vertical energies were calculated at the LR-PCM/TD-DFT (E_{LR}) and SS-PCM/TD-DFT (E_{SS}) level.

GROUND GEOMETRY (S0)						
**Functional**	**E_{LR} (nm)**	**E_{SS} (nm)**	**E_{SS}-E_{LR} (cm^{-1})**	**F_{osc}**	**Transitions**	
BLYP	479.05	468.06	490	0.3788	100→102	0.37632
					101→102	0.60289
					101←102	-0.1037
B3LYP	451.27	432.37	969	0.6502	100→102	0.15691
					101→102	0.69234
BHLYP	434.12	412.61	1201	0.7781	101→102	0.7014
CAM-B3LYP	447.34	425.37	1154	0.7386	101→102	0.69983
PBE	473.75	462.96	492	0.3849	100→102	0.37686
					101→102	0.60247
					101←102	-0.1036
PBE0	444.37	424.87	1033	0.6873	100→102	0.12924
					101→102	0.69709
M06L	443.08	428.77	753	0.5622	100→102	0.25869
					101→102	0.66166
					101←102	-0.1033
M06	451.65	430.85	1069	0.6958	100→102	0.10024
					101→102	0.70083
M062X	450.84	429.08	1125	0.7212	101→102	0.70077
M06HF	467.13	441.33	1251	0.7526	101→102	0.69649
M11L	444.76	428.65	845	0.6515	100→102	-0.1848
					101→102	0.68534
					101←102	-0.107
M11	453.75	430.36	1198	0.7582	101→102	0.69767

Table S3. Bodipy lowest energy transitions calculated with the labeled functional in benzene using the S_1 optimized geometry. Vertical energies were calculated at the LR-PCM/TD-DFT (E_{LR}) and SS-PCM/TD-DFT (E_{SS}) level.

EXCITED STATE GEOMETRY (S1)						
**Functional**	**E_{LR} (nm)**	**E_{SS} (nm)**	**E_{SS}-E_{LR} (cm^{-1})**	**F_{osc}**	**Transitions**	
BLYP	683	698	-298	0.0223	100→102	0.23616
					101→102	0.66507
B3LYP	478	461	800	0.5216	100→102	0.17947
					101→102	0.6865
BHLYP	449	426	1194	0.7435	101→102	0.70157
CAM-B3LYP	464	440	1132	0.695	101→102	0.69977
PBE	661	674	-293	0.0242	100→102	0.30259
					101→102	0.63744
PBE0	469	450	910	0.5833	100→102	0.14098
					101→102	0.69478
M06L	511	490	840	0.3025	100→102	0.31321
					101→102	0.63364
M06	472	451	998	0.6268	100→102	0.1024
					101→102	0.70076
M062X	471	448	1074	0.6579	101→102	0.70069
M06HF	487	459	1233	0.7041	101→102	0.69641
M11L	493	479	592	0.4433	100→102	-0.1998
					101→102	0.67842
M11	473	448	1179	0.7093	101→102	0.69729

Natural Transition Orbitals

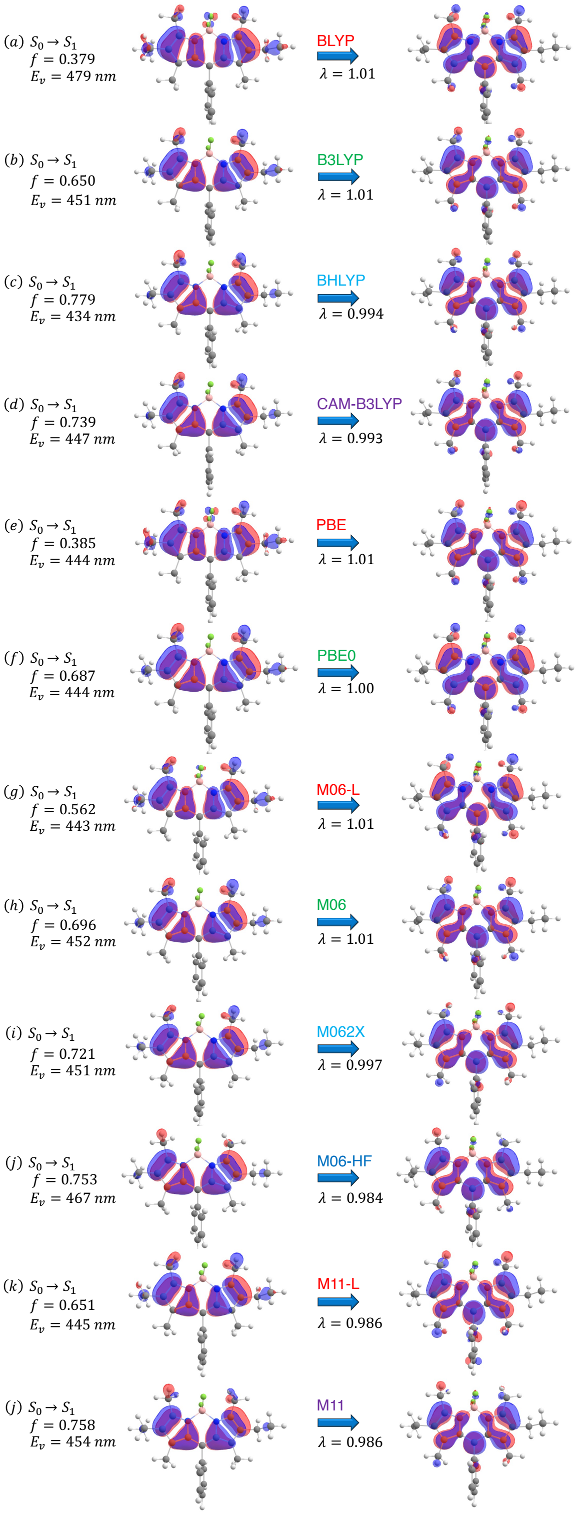

Figure S4. Natural transition orbitals of the lowest energy electronic transition in benzene using the ground state optimized geometry and labeled functionals. The oscillator strength ($\boldsymbol{f}$), vertical excitation energy ($\boldsymbol{E_v}$) and the associated eigenvalue ($\boldsymbol{\lambda}$) are presented for each transition.

RMS and MSA for each mode considered

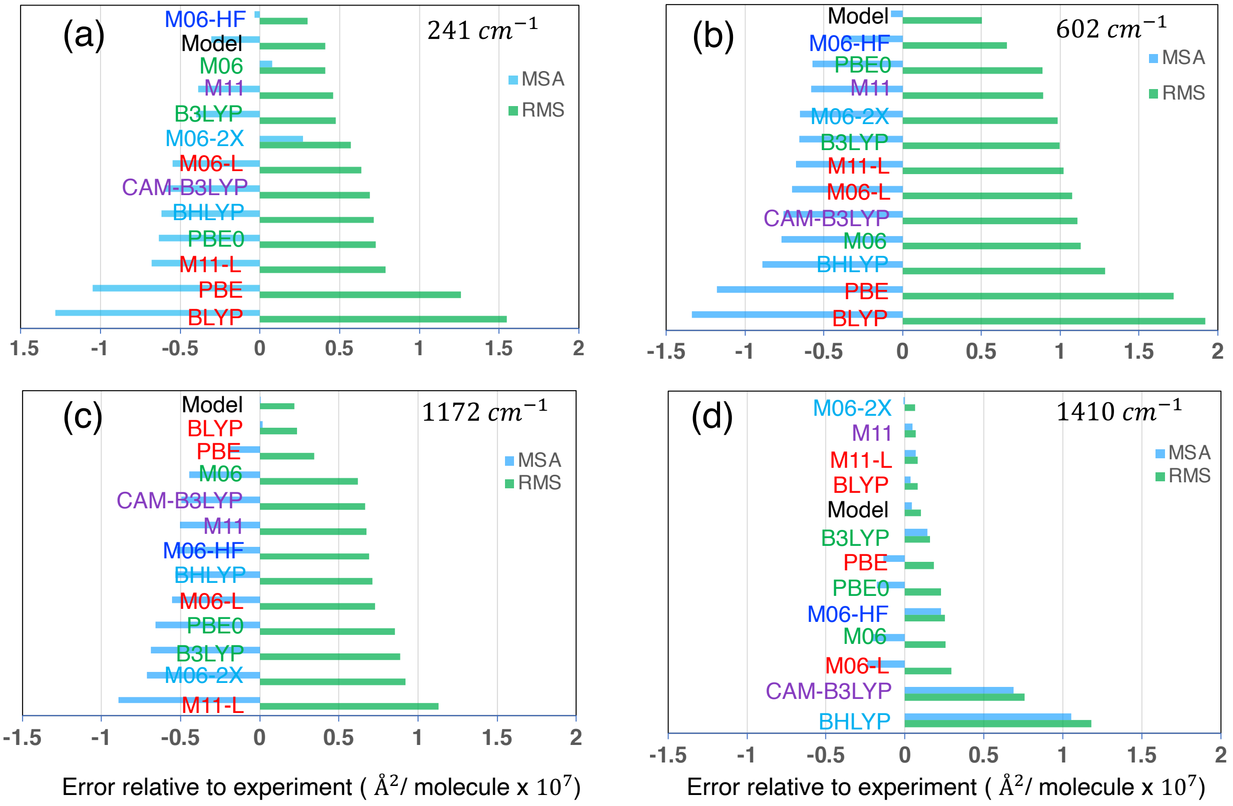

Figure S5. RMS and MSA difference (in Å^2/molecule) between the experimental and DFT calculated RR excitation profile for the four modes considered.

Additional Resonance Raman Spectra

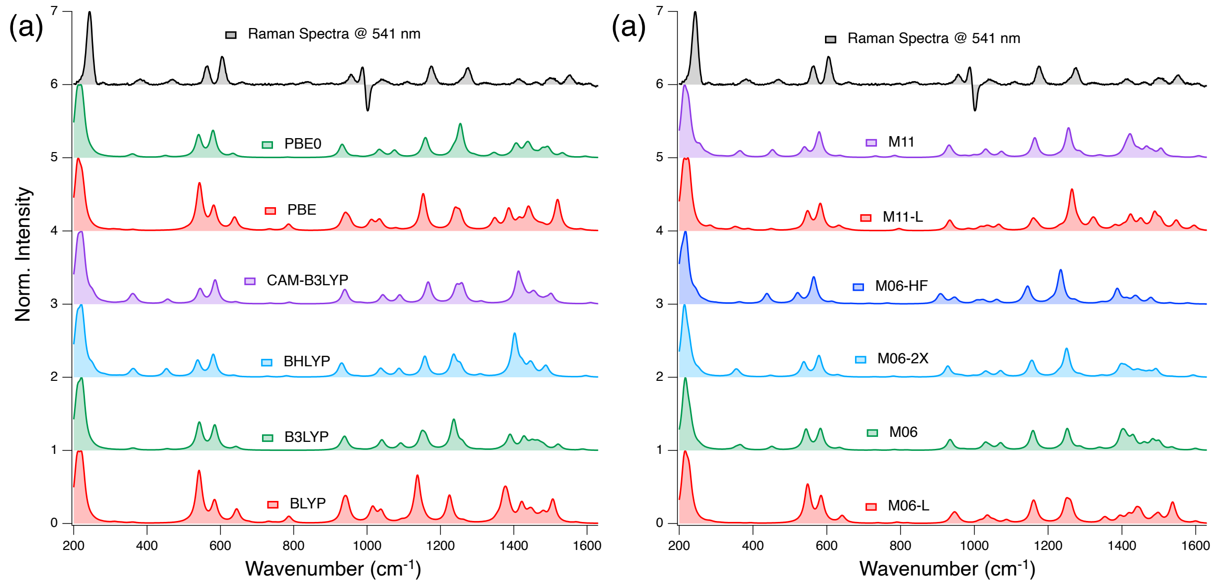

Figure S6. Experimental and DFT/TD-DFT calculated Resonance Raman Spectra at 541 nm using the labeled XCF.

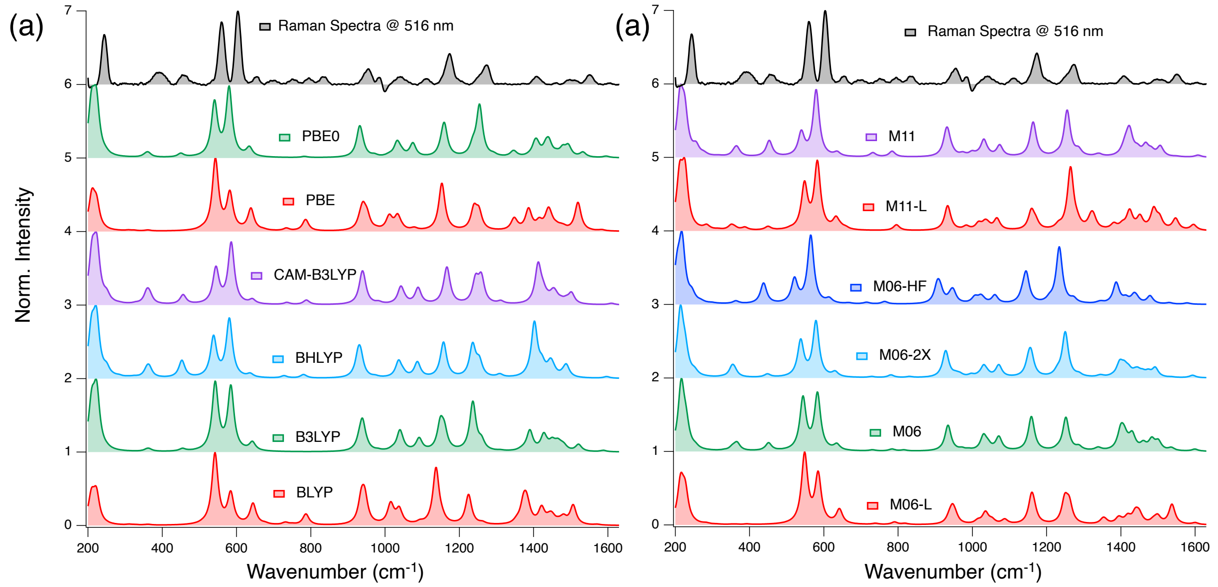

Figure S7. Experimental and DFT/TD-DFT calculated Resonance Raman Spectra at 516 nm using the labeled XCF.

Maximum overlap between the DFT calculated and experimental RR spectra at 530 nm.

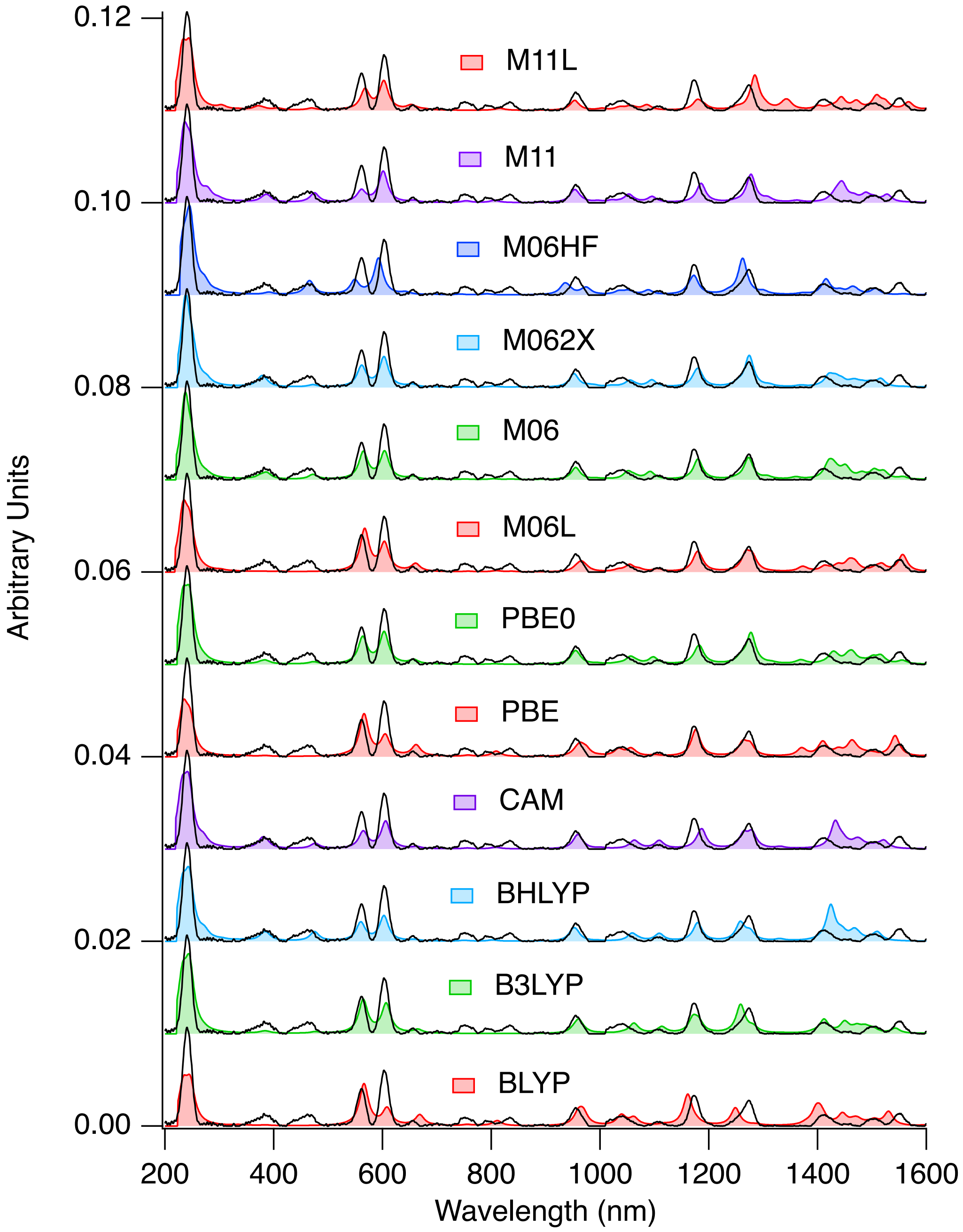

Figure S 8. Experimental (black) and labeled DFT calculated RR. The DFT calculated RR spectra have been shifted until the maximum overlap between both spectra is achieved.

Bibliography

[1] A. B. Myers and R. A. Mathies, "Resonance Raman Intensities: A Probe of Excited-State Structure and Dynamics. In Biological Applications of Raman Spectrometry: Vol. 2-Resonance Raman Spectra of Polyenes and Aromatics," *Ed. John Wiley & Sons, Inc,* 1987.

[2] A. B. Myers, R. A. Mathies, D. J. Tannor and E. J. Teller, "Excited state geometry changes from preresonance Raman intensities: Isoprene and hexatriene," *The Journal of Chemical Physics,* vol. 77, pp. 3857-3866, 1982.

[3] A. P. Shreve and R. A. Mathies, "Thermal effects in resonance raman scattering: analysis of the Raman intensities of rhodopsin and of the time-resolved Raman scattering of bacteriorhodopsin," *The Journal of Physical Chemistr,* vol. 99, no. 19, pp. 7285-7299, 1995.

[4] Z. Piontkowski and D. W. McCaman, "Excited-State Planarization in Donor–Bridge Dye Sensitizers: Phenylene versus Thiophene Bridges," *ournal of the American Chemical Society,* vol. 140, no. 35, p. 11046–11057, 2018.
